# Global, regional, and national burden of sudden infant death syndrome and the impact of COVID-19: a trend and health inequality analysis based on the global burden of disease study 2021

**DOI:** 10.3389/fped.2025.1623238

**Published:** 2025-07-25

**Authors:** Xinkuo Zheng, Meishen Liu, Xingwei Zhao, Xiuqi Xu, Wei Tao, Ling Wu, Weijia Sun, Yuhang Dong, Yalin Xi

**Affiliations:** ^1^ Department of Pharmacy, Central Hospital of Dalian University of Technology, Dalian, China; ^2^ Department of Pharmacy, The Second Affiliated Hospital of Dalian Medical University, Dalian, China; ^3^ Department of Pediatrics, Central Hospital of Dalian University of Technology, Dalian, China; ^4^ Department of Pharmacy, Dalian Women and Children’s Medical Group, Dalian, China; ^5^ Department of Neurology, People’s Liberation Army Northern Theater Command General Hospital, Shenyang, China

**Keywords:** sudden infant death syndrome, global burden of disease study, epidemiology, health inequality analysis, risk factors

## Abstract

**Background:**

Current sudden infant death syndrome (SIDS) epidemiological patterns and COVID-19 impacts remain uncertain. We therefore conducted this global, regional, and national epidemiological study using data from the Global Burden of Disease Study (GBD) 2021.

**Methods:**

This study analyzed GBD-based population data on SIDS disability-adjusted life years (DALYs). Age-standardized DALY rates (ASDR; per 100,000 population) with 95% uncertainty intervals (UIs) were calculated for 204 countries and territories, stratified by age, location, and socio-demographic index (SDI).

**Results:**

In 2021, the ASDR of SIDS accounted for 44.16 (95% UI: 25.70–59.26) per 100,000 population globally, which was a 58.97% decrease from 1990. The low and low-middle SDI quintiles exhibited a disproportionately higher disease burden of SIDS among the five SDI quintiles in 2021. Higher sociodemographic status showed an inverse association with SIDS burden, with high-SDI countries demonstrating a greater reduction compared to low-SDI counterparts from 1990 to 2021 based on age-period-cohort analysis. Although the global burden of SIDS had maintained a sustained downward trend prior to the pandemic, COVID-19 disruptions may have attenuated mitigation progress, with trend analysis suggesting a possible plateau in SIDS burden during this period rather than continued decline. Study findings indicate that although the global incidence of SIDS has shown a steady decline, persistent regional disparities underscore long-standing public health challenges.

**Conclusion:**

The burden of SIDS-related DALYs remains substantial, and its post-pandemic evolution trends necessitate dynamic tracking through robust epidemiological surveillance systems.

## 1 Introduction

Sudden infant death syndrome (SIDS), defined as the unexpected death of healthy sleeping infants under 12 months, remains the leading cause of global infant mortality beyond the neonatal period (1, 2). As a sudden unexpected infant death (SUID) subtype, SIDS cases remain unexplained even after standard autopsy, death scene review, and clinical history assessment (1). Data from the Centers for Disease Control and Prevention (CDC) showed that in 1990, the incidence of SIDS varied between 1.5 and 3 cases per 1,000 live births in most countries (3, 4). However, by 2000, this figure had decreased to approximately 0.2–1 case per 1,000 live births. Notably, in the United States (U.S.) in 2021, SIDS remained the third most common cause (7.3%) of deaths in infants under 1 year of age (3).

Despite decades of investigation, the etiology of SIDS remains incompletely understood, involving multifactorial mechanisms and absent identifiable biological precursors (5). Research suggests SIDS stems from complex interactions among genetic, environmental, and developmental factors, impeding clear understanding of its causes and disease processes (6). Established risk factors for SIDS encompass sleep position, bed-sharing, soft bedding, non-optimal sleep surfaces, smoke exposure, and prematurity (7). Furthermore, emerging evidence has implicated the prenatal period as a critical developmental window, with longitudinal studies demonstrating significant epidemiological associations between *in utero* exposure to neurotoxic substances (e.g., alcohol, cannabis, opioids) and elevated SIDS risk (8).

The COVID-19 pandemic has exerted a profound impact on global healthcare systems, with notable implications for SIDS incidence and mortality (4). A retrospective analysis of CDC mortality data indicated sustained elevation in SIDS rates during the pandemic compared to pre-pandemic baselines (9). Similarly, analysis of U.S. vital records (2015–2020) showed declining SIDS incidence from 2015 to 2019, with a sharp rise in 2020 (10). The growing number of COVID-19 cases among infants, pregnant women, and lactating mothers may have contributed to a rise in SIDS and impacted epidemiological research on the condition during the COVID-19 pandemic.

However, current epidemiological patterns of SIDS and the impacts of COVID-19 remain uncertain. Given this knowledge gap, using data from the Global Burden of Disease (GBD) 2021 study, we evaluated temporal trends in the burden of SIDS and associated risk factors between 1990 and 2021, stratified by sex and geographic region, to inform targeted prevention strategies. Additionally, an analysis focusing on the 2019–2021 period was conducted to evaluate potential disruptions attributable to the COVID-19 pandemic.

## 2 Materials and methods

### 2.1 Data source

The GBD 2021 systematically estimated global, regional, and national incidence, mortality, and disability-adjusted life years (DALYs) for 376 diseases and injuries across 204 countries and territories over a 32-year observational period (1990–2021) (11). The present analysis used de-identified aggregate data from the GBD 2021 collaborative project. As this analysis exclusively employed secondary data following standard GBD protocols, no direct participant contact occurred and no patients were involved in study design, analysis, or interpretation phases. As pre-processed data, these epidemiological estimates limit detailed documentation of uncertainty quantification methods in this study.

For our analytical metrics, we utilized DALYs data for SIDS from the GBD 2021. For SIDS, DALYs only include years of life lost (YLLs) to measure premature death burden because this condition has no disability outcomes requiring years lived with disability (YLDs) (11). We obtained SIDS data via the Global Health Data Exchange (GHDx) repository and its analytical instruments, including the Global Burden of Disease Study Results Tool (accessible at https://vizhub.healthdata.org/gbd-results/). Notably, these variables were excluded from our analytical framework due to inherent limitations in the GBD data structure, specifically the lack of documented racial/ethnic information, comorbidities, and detailed patient-level clinical characteristics.

### 2.2 Socio-demographic index

The Socio-demographic Index (SDI) synthesizes key development dimensions by integrating three standardized components through geometric mean calculation: (1) age-specific fertility rate (ages <25 years), (2) population-weighted educational attainment (ages ≥15 years), and (3) lag-adjusted per capita income (12). Using established SDI cutoffs, nations and regions were assigned to five socioeconomic development tiers: low 0–0.4658), low-middle 0.4658–0.6188), middle 0.6188–0.7120), high-middle 0.7120–0.8103), and high 0.8103–1.0000) (13). This hierarchical approach allowed us to systematically examine how socioeconomic development levels relate to population health indicators across different development stages.

### 2.3 Risk factors

GBD 2021 quantified mortality and disability resulting from 87 specific and combined risks globally, regionally, and nationally (12). This iteration employed either spatiotemporal Gaussian process regression or Disease Modeling Meta-Regression 2.1 (DisMod-MR 2.1) to model exposure levels for risk factors. The Population Attributable Fraction (PAF) represents the proportion of disease cases in a population attributable to a specific risk factor (14). It indicates the reducible fraction of disease burden achievable through risk factor elimination. PAF is commonly used in epidemiological studies to evaluate the public health impact of a risk factor on a specific disease. Evidence-based relative risks (RRs) were quantified for each risk-outcome association. These RRs were integrated with corresponding exposure estimates to compute PAFs. The analysis identified 4 risk factors associated with SIDS in the GBD 2021, covering 2 main categories: environmental/occupational risks (ambient particulate matter pollution and household air pollution from solid fuels) and behavioral risks (short gestation and low birth weight). However, certain currently known risk factors—such as maternal smoking and unsafe sleep practices—are not captured in the GBD database. Using its predefined causal inference methods, the GBD 2021 framework systematically established links between risk factors and SIDS (2).

### 2.4 Statistics

SIDS burden assessment incorporated both age-standardized rates and absolute counts of mortality and DALYs. Age-standardized rates were calculated per 100,000 population, with absolute case numbers presented as total counts, accompanied by 95% uncertainty intervals (UIs). The estimated annual percentage changes (EAPC) and its corresponding 95% confidence intervals (CIs) were derived by fitting log-linear regression models to assess temporal trends in age standardized disability-adjusted life years rate (ASDR) over the period from 1990 to 2021. A two-sided *p*-value threshold of <0.05 was adopted for assessing statistical significance. The detailed descriptions for all analytical approaches—such as the slope index of inequality, concentration index, frontier analysis, decomposition analysis, and Bayesian age-period-cohort modeling—are fully elaborated in subsequent methodological chapters. Analytical workflows were implemented through JD_GBDR software (v2.36, Jingding Medical Technology Co., Ltd) and R statistical environment (v4.3.2).

### 2.5 Cross-country inequality analysis

Health inequality metrics including the Slope Index of Inequality (SII) and Concentration Index of Inequality (CII) assessed SDI-associated disparities in SIDS burden across nations (15). The SII was quantified through weighted least squares regression analysis, modeling the relationship between DALYs rates and population-weighted SDI ranks derived from cumulative distribution midpoints of nationally stratified SDI quintiles (16). We conducted a longitudinal assessment of health inequality dynamics across 204 countries and territories between 1990 and 2021. The CII used an economics-based method to build Lorenz-type curves. These curves plot cumulative population proportions in SDI quintiles against corresponding SIDS burden distributions, with inequality levels measured by numerically integrating the curve deviations.

### 2.6 Frontier analysis

To evaluate the association between the burden of SIDS and sociodemographic development levels, we utilized frontier analysis to develop an ASDR-based frontier model using SDI as the primary predictor. Unlike standard regression models that examine variable relationships or predict outcomes, we applied advanced statistical methods like frontier analysis. This approach addressed the non-linear link between SDI and disease burden, while identifying multidimensional factors contributing to SIDS burden. Frontier analysis aims to estimate the theoretically achievable minimum ASDR for each country or territory based on its current development level, establishing a benchmark for optimal performance (17). This methodology measures the gap between observed disease burden and attainable minimum burden, identifying priority regions for intervention. Our analytical framework applied locally weighted scatterplot smoothing (LOESS) combined with local polynomial regression, utilizing adjustable smoothing parameters (0.30, 0.40, 0.50) to develop optimized frontier curves that accurately model the non-linear SDI-ASDR association. To enhance methodological rigor, we conducted 1,000 bootstrap replications and derived mean ASDR values corresponding to each SDI level. By calculating the absolute gap (effective difference) between each country/territory's 2021 ASDR values and the optimal frontier curve, we estimated geographically achievable reduction potential (18).

## 3 Results

### 3.1 Global burden of SIDS

**Figure 1:**
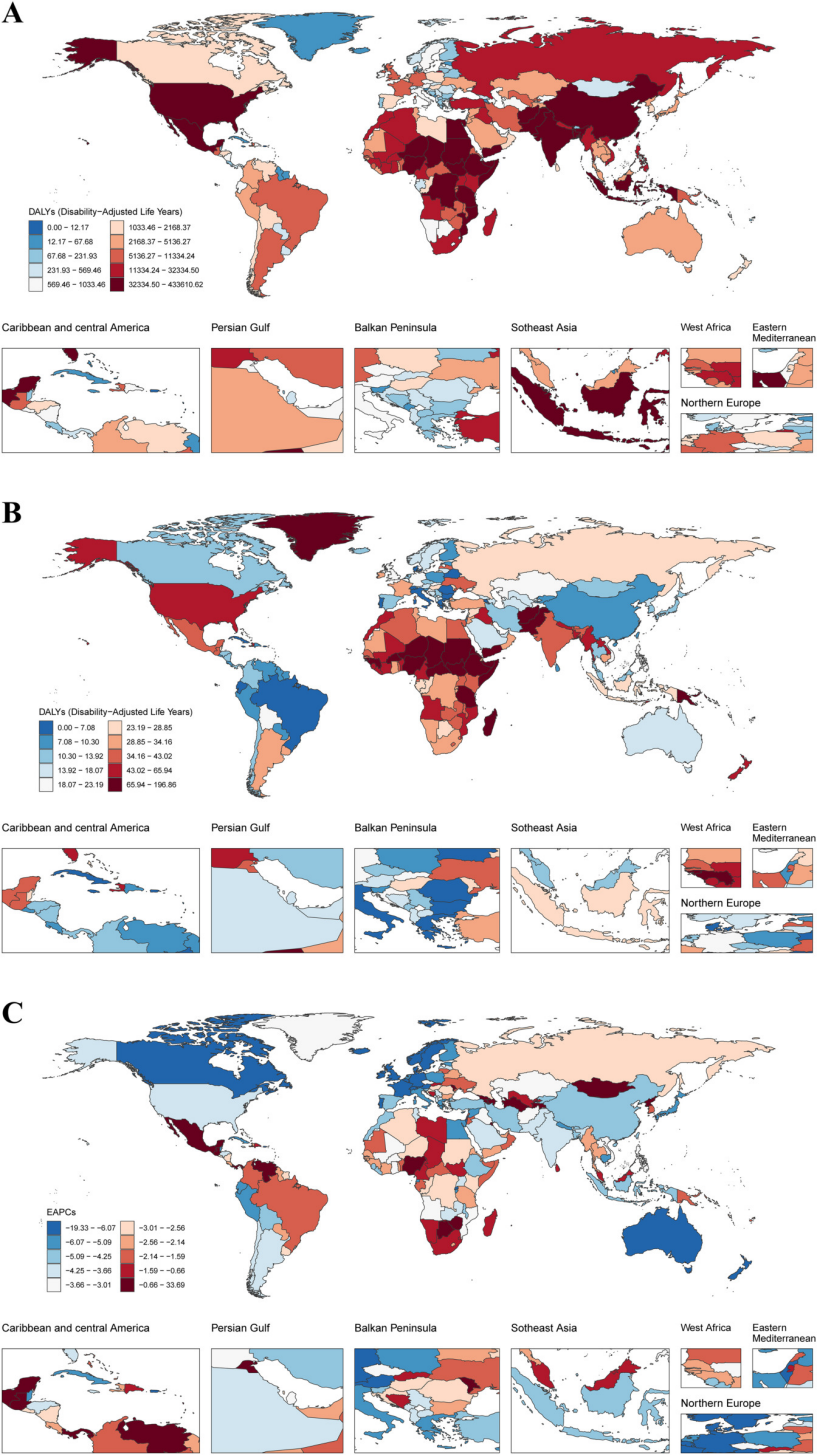
DALYs of SIDS across 204 countries and territories. **(A)** DALYs number in 2021 **(B)** ASDR in 2021 **(C)** EAPC between 1990 and 2021. DALYs, disability-adjusted life years; SIDS, sudden infant death syndrome; ASDR, age standardized Dalys rate; EAPC, estimated annual percentage changes.

Between 1990 and 2021, SIDS-associated DALYs for both sexes combined declined significantly by 59.58%, with absolute values decreasing from 6,794,659.76 (95% UI: 4,121,026.03–10,289,656.44) to 2,746,174.49 (95% UI: 1,598,180.42–3,686,289.79). Sex-specific analysis showed concordant declines, with females experiencing a 62.94% reduction from 3,399,486.70 (95% UI: 1,611,240.58–5,663,274.55) to 1,259,805.70 (95% UI: 585,867.02–1,752,413.44), while males demonstrated a 56.22% decrease from 3,395,173.06 (95% UI: 1,788,727.10–5,364,023.52) to 1,486,368.80 (95% UI: 721,571.21–2,241,382.43). Notably, the female-to-male disparity persisted throughout the study period, evidenced by a stable sex ratio of 1.00 in 1990 vs. 0.85 in 2021 (Supplementary Table S1; Figure 1). Correspondingly, the ASDR for both sexes combined declined significantly by 58.97%, with values decreasing from 107.64 (95% UI: 65.31–162.96) per 100,000 population to 44.16 (95% UI: 25.70–59.26) per 100,000 population. Sex-specific analysis showed consistent declines, with females experiencing a 62.94% reduction from 111.43 (95% UI: 52.84–185.57) per 100,000 population to 41.91 (95% UI: 19.49–58.29) per 100,000 population, while males demonstrated a 56.22% decrease from 104.10 (95% UI: 54.85–164.45) per 100,000 population to 46.26 (95% UI: 22.46–69.76) per 100,000 population (Table 1; Figure 1).

**Table 1:** Global and regional ASDR of SIDS in 1990 and 2021, EAPCs of 1990–2021 and 2019–2021.

Location	ASDR per 100,000 population (95% UI)		EAPC of ASDR (95% CI)	
	1990	2021	1990–2021	2019–2021
Global	107.64 (65.31–162.96)	44.16 (25.70–59.26)	-3.00 (−3.16 to −2.83)	−.26 (−24.40 to 18.74)
Sex				
Female	111.43 (52.84–185.57)	41.91 (19.49–58.29)	−3.30 (−3.53 to −3.07)	−5.93 (−27.59 to 22.21)
Male	104.10 (54.85–164.45)	46.26 (22.46–69.76)	−2.71 (−2.82 to −2.60)	−4.67 (−21.54 to 15.82)
SDI				
Low SDI	172.81 (84.81–275.36)	77.75 (40.48–113.81)	−2.63 (−2.84 to −2.43)	−5.45 (−20.00 to 11.76)
Low-middle SDI	147.96 (71.99–268.96)	44.92 (24.67–66.38)	−3.89 (−4.12 to −3.65)	−9.16 (−40.10 to 37.74)
Middle SDI	49.70 (29.32–71.19)	20.70 (12.87–28.66)	−2.74 (−2.81 to −2.68)	−6.07 (−23.53 to 15.37)
High-middle SDI	42.89 (31.21–60.44)	16.42 (11.58–21.27)	−3.48 (−3.67 to −3.29)	1.60 (−19.21 to 27.78)
High SDI	162.80 (157.16–168.21)	32.22 (28.12–36.47)	−4.94 (−5.18 to −4.70)	−4.16 (−11.38 to 3.64)
Region				
Andean Latin America	45.86 (21.89–80.37)	9.99 (5.71–16.30)	−5.18 (−5.57 to −4.79)	−7.99 (−23.98 to 11.37)
Australasia	317.60 (297.70–338.19)	24.41 (18.19–31.67)	−7.47 (−7.77 to −7.17)	−9.14 (−15.91 to −1.82)
Caribbean	36.91 (19.69–72.16)	25.01 (11.03–47.69)	−1.41 (−1.65 to −1.18)	−0.98 (−7.74 to 6.27)
Central Asia	21.89 (8.52–32.71)	16.55 (10.59–25.75)	−1.11 (−1.30 to −0.91)	−3.07 (−10.68 to 5.18)
Central Europe	33.82 (27.23–41.34)	10.92 (7.59–15.43)	−3.96 (−4.23 to −3.69)	−4.76 (−5.73 to −3.78)
Central Latin America	23.72 (20.64–27.62)	25.62 (18.85–34.87)	0.88 (0.49–1.27)	−6.70 (−30.80 to 25.78)
Central Sub-Saharan Africa	89.41 (30.99–193.32)	36.76 (15.68–70.28)	−2.83 (−3.06 to −2.59)	−5.65 (−7.08 to −4.19)
East Asia	27.24 (15.06–42.80)	9.01 (3.92–15.03)	−4.76 (−5.39 to −4.13)	18.19 (−75.61 to 472.68)
Eastern Europe	51.29 (43.83–61.20)	26.60 (22.17–33.92)	−2.59 (−3.03 to −2.14)	3.83 (−18.26 to 31.88)
Eastern Sub-Saharan Africa	174.94 (84.89–284.66)	63.49 (34.16–93.93)	−3.34 (−3.67 to −3.01)	−5.14 (−16.32 to 7.53)
High-income Asia Pacific	33.84 (26.17–44.55)	11.15 (7.90–14.69)	−4.42 (−4.86 to −3.98)	−6.53 (−12.53 to −0.12)
High-income North America	224.05 (214.74–232.83)	54.30 (46.03–62.55)	−4.16 (−4.45 to −3.87)	−4.28 (−4.55 to −4.00)
North Africa and Middle East	176.79 (103.56–266.97)	62.66 (35.92–94.27)	−3.40 (−3.54 to −3.25)	−5.33 (−18.56 to 10.05)
Oceania	95.72 (33.48–181.97)	61.71 (28.47–106.46)	−1.65 (−1.90 to −1.39)	−2.38 (−2.54 to −2.21)
South Asia	154.98 (66.95–298.16)	44.06 (23.44–69.17)	−4.14 (−4.45 to −3.82)	−11.61 (−53.58 to 68.31)
Southeast Asia	72.09 (30.99–122.51)	25.96 (11.27–38.82)	−3.55 (−3.69 to −3.40)	−2.40 (−15.60 to 12.86)
Southern Latin America	97.87 (72.52–130.17)	28.21 (20.65–38.15)	−3.78 (−4.43 to −3.13)	−9.80 (−48.08 to 56.71)
Southern Sub-Saharan Africa	46.02 (17.72–102.28)	32.92 (12.35–78.37)	−0.99 (−1.19 to −0.78)	−2.51 (−27.58 to 31.24)
Tropical Latin America	11.01 (9.26–12.94)	6.36 (4.91–8.06)	−1.71 (−2.36 to −1.06)	−9.86 (−45.31 to 48.58)
Western Europe	171.07 (163.57–178.77)	18.38 (15.40–21.91)	−6.85 (−7.25 to −6.44)	−6.00 (−33.32 to 32.53)
Western Sub-Saharan Africa	130.75 (65.30–204.09)	84.56 (41.35–127.41)	−1.18 (−1.28 to −1.07)	−5.49 (−17.46 to 8.21)

ASDR, age standardized disability-adjusted life years rate; SIDS, sudden infant death syndrome; EAPC, estimated annual percentage changes; UI, uncertainty intervals; CI, confidence interval; SDI, socio-demographic index.

From 1990 to 2021, the global SIDS-related ASDR for both sexes combined showed a significant decline, with an EAPC of −3.00 (95% CI: −3.16 to −2.83). Sex-stratified analyses revealed parallel downward trends, with female-specific EAPC reaching −3.30 (95% CI: −3.53 to −3.07) and male-specific EAPC at −2.71 (95% CI: −282 to −2.60). However, during the COVID-19 pandemic period (2019–2021), the global ASDR trend stabilized, demonstrating a non-significant EAPC of −5.26 (95% CI: −24.40 to 18.74). Sex-stratified analyses revealed parallel stabilized trends, with female-specific EAPC reaching −5.93 (95% CI: −27.59 to 22.21) and male-specific EAPC at −4.67 (95% CI: −21.54 to 15.82), both confidence intervals overlapping zero (Table 1; Figure 1).

### 3.2 SDI regional burden of SIDS

Between 1990 and 2021, all five SDI quintiles demonstrated a consistent decline in SIDS-related DALYs. The High SDI quintile showed the greatest reduction with DALYs decreasing by 83.60% from 987,580.13 (95%UI: 953,398.32–1,020,384.60) to 162,001.17 (95%UI: 141,386.97–183,364.52), while the Low SDI quintile demonstrated the smallest decline of 24.71%, decreasing from 1,761,821.05 (95%UI: 864,011.55–2,809,316.20) to 1,326,431.33 (95%UI: 690,406.38–1,942,045.37). In 2021, among the five SDI quintile groups, the low SDI group exhibited the highest ASDR of 77.75 (95%UI: 40.48–113.81) per 100,000 population, while the high-middle SDI group demonstrated the lowest ASDR of 16.42 (95%UI: 11.58–21.27) per 100,000 population. (Supplementary Tables S1, S2, Supplementary Figure S1).

From 1990 to 2021, all SDI quintile groups experienced consistent declines in SIDS-related ASDR, characterized by EAPC values of −2.63 (95% CI: −2.84 to −2.43) for Low SDI, −3.89 (95% CI: −4.12 to −3.65) for Low-middle SDI, −2.74 (95% CI: −2.8 to −2.68) for Middle SDI, −3.48 (95% CI: −3.67 to −3.29) for High-middle SDI, and −4.94 (95% CI: −5.18 to −4.70) for High SDI. However, during the COVID-19 pandemic period (2019–2021), stabilization patterns emerged in ASDR trends across SDI quintiles, with non-significant EAPC values of −5.45 (95% CI: −20.00 to 11.76) for Low SDI, −9.16 (95% CI: −40.10 to 37.74) for Low-middle SDI, −6.07 (95% CI: −23.53 to 15.37) for Middle SDI, −1.60 (95% CI: −19.21 to 27.78) for High-middle SDI, and −4.16 (95% CI: −11.38 to 3.64) for High SDI (Table 1).

### 3.3 Geographic regional burden of SIDS

**Figure 2:**
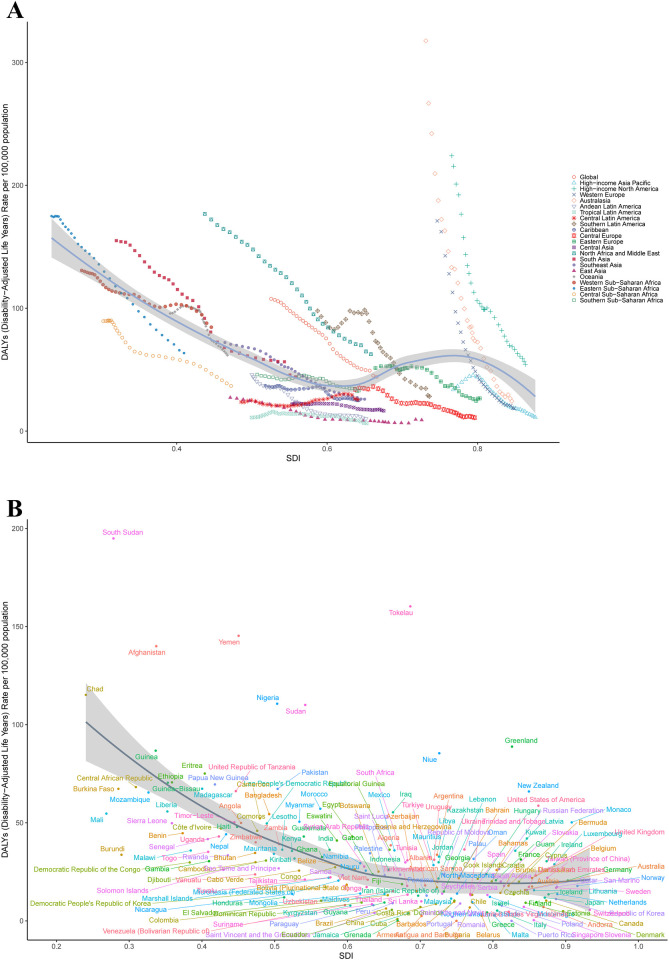
The ASDR of SIDS by SDI in 2021. **(A)** For global and 21 geographic regions, **(B)** For 204 countries and territories. ASDR, age standardized disability-adjusted life years rate; SIDS, sudden infant death syndrome; SDI, socio-demographic index.

Between 1990 and 2021, among the 21 GBD regions, 19 exhibited reductions in DALYs. The most pronounced decrease occurred in Australasia, where DALYs declined from 48,727.34 (95%UI: 45,673.82–51,887.08) to 4,245.98 (95%UI: 3,164.88–5,509.85), reflecting a 91.29% reduction. In contrast, Central Latin America demonstrated the smallest decline, with DALYs decreasing from 56,415.45 (95%UI: 49,086.69–65,707.31) to 48,620.12 (95%UI: 35,751.39–66,227.66), equivalent to a 13.82% decrease. Notably, Oceania and Western Sub-Saharan Africa were the only two regions with DALYs increases, showing rises of 23.62% and 31.38%, respectively. In 2021, among the 21 GBD regions, Western Sub-Saharan Africa exhibited the highest ASDR of 84.56 (95%UI: 41.35–127.41) per 100,000 population, while Tropical Latin America demonstrated the lowest ASDR of 6.36 (95%UI: 4.91–8.06) per 100,000 population (Supplementary Table S1; Figure 2).

From 1990 to 2021, SIDS-related ASDR significantly declined in 20 of 21 GBD regions. Australasia demonstrated the steepest reduction with an EAPC of −7.47 (95% CI: −7.77 to −7.17), while Southern Sub-Saharan Africa showed the smallest decrease at an EAPC of −0.99 (95% CI: −1.19 to −0.78). In contrast, Central Latin America exhibited an upward trend with an EAPC of 0.88 (95% CI: 0.49–1.27). However, during the COVID-19 pandemic period (2019–2021), only 6 of 21 GBD regions showed significant ASDR declines based on EAPC values, while 15 regions demonstrated stable trends (Table 1).

### 3.4 National burden of SIDS

Between 1990 and 2021, DALYs declined in 179 of 204 countries globally, while 24 countries demonstrated increases. Only one country maintained stable DALYs throughout this period (Antigua and Barbuda). In 2021, the five countries with the highest DALYs were Nigeria at 429,317.45 (95% UI: 186,071.91–728,042.61), India at 381,394.89 (95% UI: 187,003.25–628,218.34), Pakistan at 198,314.80 (95% UI: 89,410.51–371,961.65), Ethiopia at 116,634.41 (95% UI: 56,320.99–206,189.41), and the United States of America at 105,314.81 (95% UI: 89,369.39–121,542.28). Conversely, the highest national ASDRs were observed in South Sudan at 194.91 (95% UI: 90.51–359.28) per 100,000 population, Tokelau at 160.29 (95% UI: 70.52–292.92) per 100,000 population, Yemen at 145.29 (95% UI: 65.29–292.93) per 100,000 population, Afghanistan at 139.98 (95% UI: 68.96–237.06) per 100,000 population, and Chad at 115.16 (95% UI: 51.59–207.66) per 100,000 population (Supplementary Tables S1, S3; Figure 2).

From 1990 to 2021, significant reductions in SIDS-related ASDR were observed in 187 nations; conversely, 9 countries experienced notable increases, and 12 countries maintained stable patterns according to EAPC analyses. However, during the COVID-19 pandemic period (2019–2021), significant reductions in SIDS-related ASDR were observed in 59 nations; conversely, 1 country experienced notable increases, and 144 countries maintained stable patterns (Supplementary Table S3).

### 3.5 DALYs of SIDS attribute to risk factors

**Figure 3:**
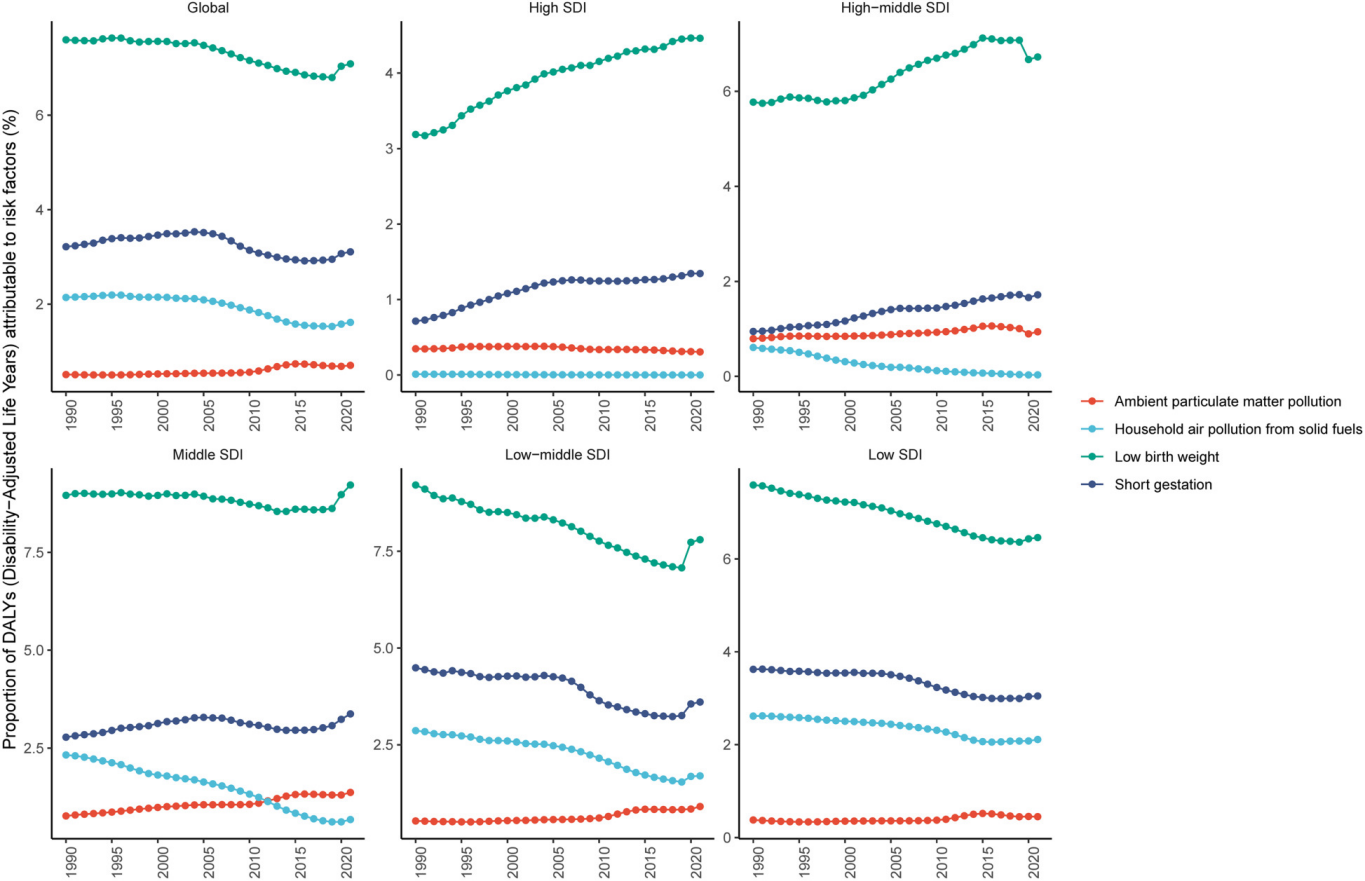
Changes in proportion of DALYs attributable to risk factors of SIDS for global and five SDI regions, 1990–2021. DALYs, disability-adjusted life years; SIDS, sudden infant death syndrome; SDI, socio-demographic index.

This study identified four modifiable risk factors significantly associated with SIDS: ambient particulate matter pollution, household air pollution from solid fuels, short gestation, and low birth weight. Globally, low birth weight was the leading risk factor, with a PAF (%) of 7.08 (95% UI: 5.89–8.45) in 2021. Other risk factors included short gestation with a PAF (%) of 3.11 (95% UI: 2.51–3.73), household air pollution from solid fuels at 1.62 (95% UI: 1.19–2.10), and ambient particulate matter pollution at 0.71 (95% UI: 0.46–1.00). From 1990 to 2021, the proportion of DALYs attributable to key risk factors varied significantly across SDI quintiles. The disease burden of SIDS caused by low birth weight exhibited an upward trend in high and high-middle SDI quintiles. Similarly, the burden associated with short gestation increased significantly in high, high-middle, and middle SDI quintiles. In contrast, ambient particulate matter pollution-related health impacts remained relatively stable across all SDI categories during the study period. Notably, household air pollution from solid fuels demonstrated a declining trend in all regions except for high-SDI areas (Figure 3; Supplementary Table S4).

In 2021, among 21 GBD regions, Andean Latin America exhibited the highest proportion of SIDS-associated DALYs attributable to ambient particulate matter pollution, with a PAF (%) of 1.64 (95% UI: 0.68–2.98). Southeast Asia showed the highest proportion of DALYs linked to low birth weight, with a PAF (%) of 14.06 (95% UI: 7.60–19.18). The Caribbean had the highest proportion of DALYs associated with household air pollution from solid fuels and short gestation, with PAF values (%) of 3.68 (95% UI: 1.77–6.35) and 6.76 (95% UI: 4.04–10.22), respectively (Supplementary Figure S2, Supplementary Table S4).

### 3.6 Cross-country inequality analysis

**Figure 4:**
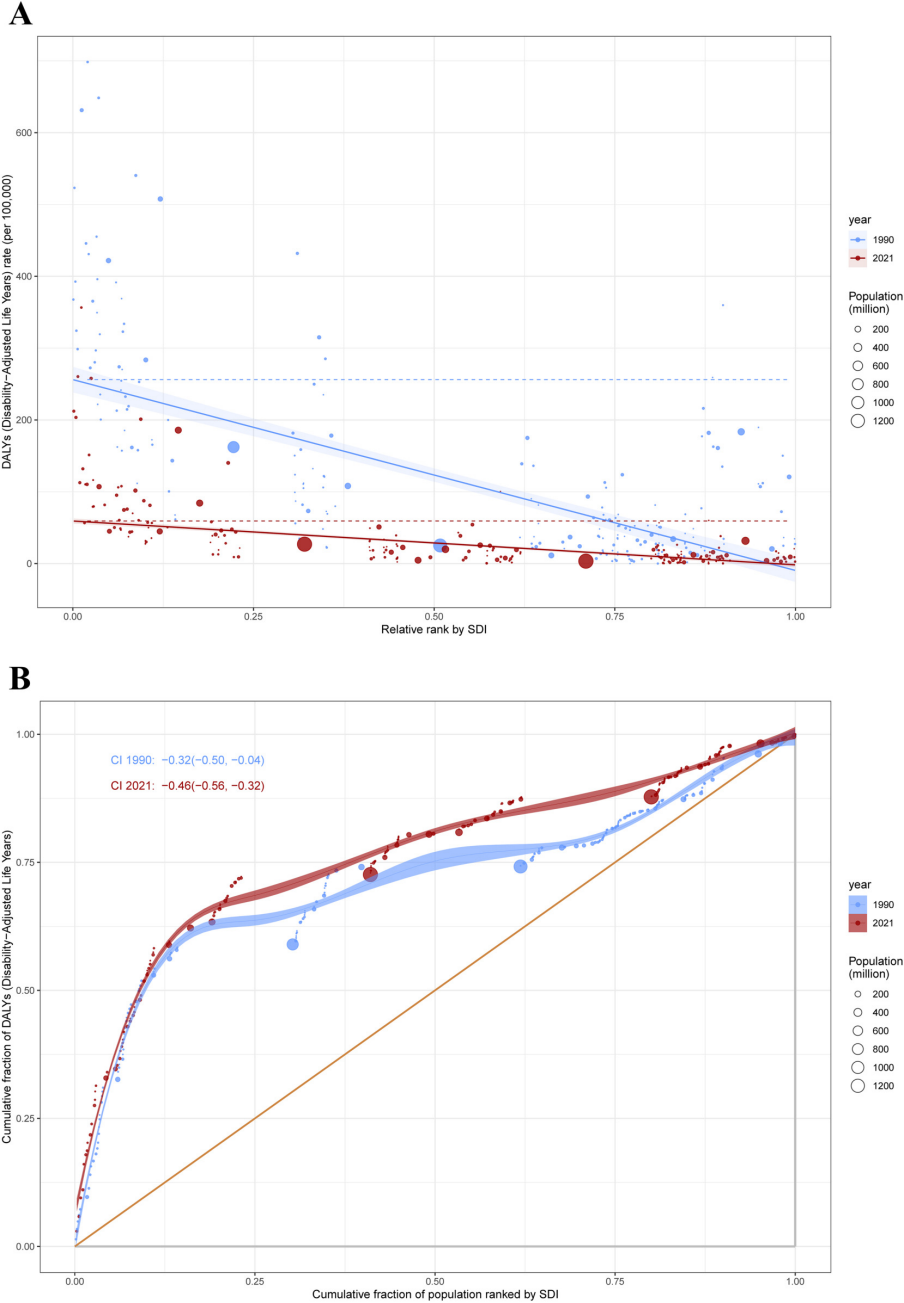
Health inequality regression curves **(A)** and concentration curves **(B)** for the DALYs of SIDS. DALYs, disability-adjusted life years; SIDS, sudden infant death syndrome; SDI, socio-demographic index.

Our analysis of SIDS burden revealed pronounced disparities in both absolute and relative terms across the SDI gradient. Low-SDI countries and territories demonstrated disproportionately elevated disease burdens compared to their high-SDI counterparts. The slope index of inequality demonstrated a narrowing disparity in DALYs rates between extreme SDI quintiles, declining from −265.78 (95% CI: −297.58 to −233.98) in 1990 to −61.08 (95% CI: −68.40 to −53.76) by 2021. Notably, the concentration index intensified from −0.32 (95% CI: −0.50 to −0.04) in 1990 to −0.46 (95% CI: −0.56 to −0.32) in 2021. This pattern demonstrates divergent trends: while absolute inequality in SIDS burden diminished over the three-decade period, relative inequality measures exhibited significant amplification (Figure 4).

### 3.7 Frontier analysis

**Figure 5:**
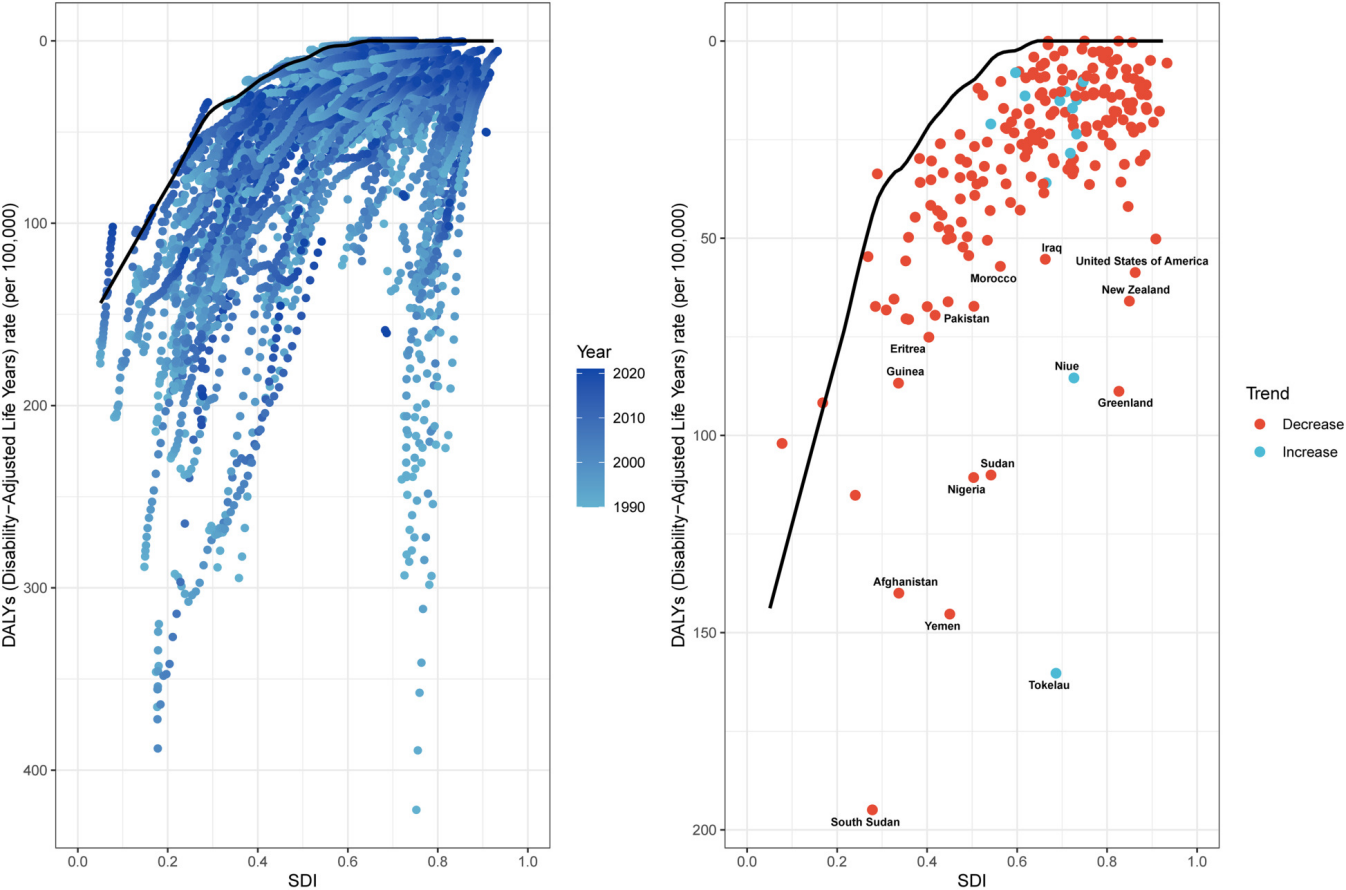
Frontier analysis exploring the relationship between SDI and ASDR for SIDS, 1990–2021. ASDR, age standardized disability-adjusted life years rate; SIDS, sudden infant death syndrome; SDI, socio-demographic index.

Frontier analysis employing ASDR and SDI metrics (1990–2021) quantified the theoretically achievable reduction potential for SIDS burden across development spectrums. This methodology established optimal performance benchmarks by evaluating ASDR trends relative to national/regional socioeconomic development tiers. The 15 countries and territories with the largest actual differences in potential improvement (effective difference range: 160.27–52.73) include Tokelau, South Sudan, Yemen, Afghanistan, Sudan, Nigeria, Greenland, Niue, New Zealand, United States of America, Pakistan, Iraq, Morocco, Eritrea, and Guinea. In contrast, the 15 countries and territories with the smallest disparities in potential improvement in ASDR (effective difference range: 0.00–3.83) include Antigua and Barbuda, Burundi, Niger, Somalia, Puerto Rico, Grenada, Singapore, Cuba, Honduras, Portugal, Armenia, Belarus, Malta, Romania, and Jamaica. Frontier analysis uncovered different optimization opportunities for SIDS burden mitigation among nations at varying developmental stages. Paradoxically, select resource-constrained low-SDI countries and territories (exemplified by South Sudan, Yemen, and Afghanistan) manifested disproportionately favorable age-standardized control metrics relative to their socioeconomic positioning (Figure 5; Supplementary Table S5).

## 4 Discussion

### 4.1 Interpretation of main findings

SIDS remains a critical global public health challenge, imposing substantial disease burden on infant survival and requiring sustained research efforts (19). Our study analyzed 32 years of population-level data (1990–2021) encompassing infants under 1 year from 204 countries and territories, stratified across five SDI regions and 21 GBD regions. Using multifactorial analytical methods, we systematically characterized disease burden variations among populations, temporal phases, and geographic distributions. This study employed an innovative approach using GBD 2021 data to assess temporal trends, quantify health inequities, identify critical transition points in SIDS indicators, and examine associations between socioeconomic development levels and cross-national burden disparities. Conventional analytical frameworks demonstrated limitations in addressing the nonlinear, multivariable, and high-dimensional nature of GBD datasets. To overcome these constraints, we implemented advanced methodological approaches to ensure rigorous data interpretation. The findings advance the epidemiological understanding of SIDS dynamics and provide evidence-based insights for formulating equitable public health policies.

Our analysis revealed a consistent global reduction in SIDS burden across males, females, and both sexes combined from 1990 to 2021. This decline reflects combined improvements in diagnostic technologies, therapeutic strategies, risk factor management, and targeted screening of high-risk populations, consistent with existing epidemiological evidence (20, 21). Despite progress, persistently high mortality rates underscore how global SIDS burden is disproportionately increased by health disparities rooted in unequal healthcare access and insufficient health education. Of particular significance, the previously sustained downward trend of SIDS disease burden plateaued during the COVID-19 pandemic period. This transition suggests that the pandemic may have partially counteracted progress in SIDS mitigation, potentially exacerbating its global disease burden. Notably, this observed plateau represents a significant epidemiological anomaly. It likely stems from synergistic disruptions across three domains: healthcare fragmentation (manifested through suspended well-baby visits, resource diversion, and disrupted educational programs), exacerbated maternal stressors (including economic precarity elevating anxiety biomarkers and attenuated support networks), and systemic neonatal care delays (encompassing critical care avoidance, monitoring technology shortages, and deferred immunizations). Collectively, these cascading disruptions attenuated decades of SIDS prevention progress—disproportionately impacting vulnerable populations while revealing critical vulnerabilities in global infant health safeguards.

### 4.2 Region-specific risk factor hierarchies

Marked disparities in attributable DALYs emerged across SDI quintiles, with the high SDI group exhibiting an 8.19-fold greater burden compared to the low SDI quintile, despite comparable population sizes (1,094,047,736 vs. 1,117,382,591; GBD 2021). This difference likely reflects systemic underreporting in lower SDI regions due to incomplete disease surveillance systems, though inherently lower SIDS burden in these areas remains a plausible explanation. Low birth weight emerged as the predominant risk factor for SIDS across all SDI quintiles. However, the relative impact ranking of secondary risk factors varied significantly by development level. Household air pollution from solid fuels constituted the least influential factor in high and high-middle SDI quintiles, whereas ambient particulate matter pollution exhibited minimal impact in low and low-middle SDI quintiles. This consistent pattern of risk differences across factors highlights how regional environmental and socioeconomic conditions critically shape SIDS-related DALY distributions.

Regarding risk factors trends associated with SIDS, regional heterogeneity is evident. Behavioral risk factors have the most significant impact on SIDS, with two key contributors being low birth weight and short gestation. The five regions most significantly impacted by the low birth weight are Southeast Asia, Central Latin America, the Caribbean, Andean Latin America, and Tropical Latin America. Correspondingly, the five regions most significantly impacted by the short gestation are the Caribbean, Southeast Asia, South Asia, Oceania, and Tropical Latin America. Findings from a study utilizing the GBD 2019 data further confirmed that preterm birth and low birth weight were among the primary contributors to SIDS (22). Studies have also indicated that very low birth weight or very preterm infants are associated with an elevated risk of infant pneumonia, higher hospitalization rates, and serve as one of the contributing factors to infant mortality at home (23). Additionally, studies have demonstrated that SIDS is strongly associated with suboptimal infant sleep environments (e.g., soft bedding, overheating) and unsafe sleep practices, such as prone sleeping, bed-sharing, or placement on non-approved sleep surfaces (24, 25).

### 4.3 Health inequalities across nations and regions

As the field of global health advances, evaluating the complex nature of health disparities among nations and regions has grown in critical importance. Conventional linear regression approaches are limited in their ability to address this intricacy. Although these models can identify differences between population subgroups, they frequently fail to account for the nuanced distribution of inequalities stratified by sociodemographic variables. Conversely, the WHO-endorsed SII and CII enhance measurement precision by integrating population-weighted adjustments and subgroup hierarchical positioning (26, 27). These methods allow detailed inequality assessments, making them well-suited for the GBD 2021 dataset. Significant geographic and developmental differences in this dataset require advanced analytical frameworks to reliably measure disparities (26–28).

Income remains a central determinant of population health, with its unequal distribution significantly influencing health disparities. Higher income levels typically correlate with greater access to healthcare services and improved health outcomes. Compared to low-medium SDI countries, high-SDI nations possess more advanced medical infrastructure, more comprehensive healthcare policies, and better-trained healthcare professionals. Our analysis demonstrates that between 1990 and 2021, SIDS-related health inequalities displayed contrasting trends: absolute disparities in global disease burden decreased, while relative disparities increased. This pattern suggests that medical progress has predominantly benefited high-SDI countries, where advanced technologies and efficient healthcare systems effectively reduce disease burden. Conversely, low SDI regions show limited improvements due to insufficient medical resources and inadequate health service coverage. To achieve global health equity, we must prioritize fair medical resource distribution, increase support for low-SDI regions, and strengthen essential healthcare systems worldwide. Monitoring disease burden trends and health disparities enables policymakers to optimize resource allocation, improve health education, and implement targeted interventions, particularly in high-burden populations (29).

### 4.4 Implications for policy

To evaluate the potential optimization capacity for SIDS across 204 countries and territories, frontier analysis was implemented. This method advances beyond conventional regression analysis of variable correlations by estimating region-specific minimum achievable disease burden relative to SDI levels. By measuring the disparity between current burden levels and the “frontier” threshold of ideal outcomes, this methodology aids in identifying underperforming regions. Traditional linear approaches, however, focus primarily on existing variable correlations and frequently miss potential enhancements due to their omission of SDI-linked performance benchmarks. South Sudan, Yemen, and Afghanistan consistently emerged as low SDI regions demonstrating remarkable efficacy in reducing disease burden. Despite resource constraints, these areas have achieved significant success in mitigating SIDS through localized neonatal care initiatives and culturally tailored health education strategies, warranting further investigation into their policy frameworks. Conversely, high SDI quintiles including Puerto Rico, Singapore, and the United States Virgin Islands exhibited suboptimal performance in SIDS control. While excelling in neonatal screening and hospital interventions, improved coordination across pediatric, public health, and social care disciplines is essential to deliver sustained post-discharge monitoring and family-centered support for high-risk infants. Geographical disparities in environmental exposures, nutritional access, healthcare availability, and potential genetic susceptibility underscore the imperative for these regions to refine health policy design and execution.

### 4.5 Future research directions

Building on these findings, future research should prioritize identifying key determinants of SIDS burden and its modifying factors in diverse population groups including sex-specific categories, infant age cohorts, and socioeconomic contexts to uncover underlying mechanisms. These insights could inform evidence-based public health strategies, particularly in low SDI settings, where targeted investments in healthcare infrastructure, community health education, and resource allocation are critical to mitigating disease burden disparities. Global efforts to combat SIDS necessitate enhanced cross-border collaboration and transparent data-sharing frameworks. Although enhanced screening protocols and therapies have lowered global SIDS incidence, persistent challenges necessitate united global action. Establishing standardized lifecycle care models for at-risk infants, coupled with shared best practices and multidisciplinary resource pooling, could accelerate progress in global health equity. Future research should also explore integrating emerging technologies—such as AI-driven diagnostics, telemedicine platforms, and interoperable health record systems—into SIDS prevention programs. Such innovations hold transformative potential for low- and middle-income countries by improving diagnostic precision, streamlining resource distribution, and addressing systemic inequities in healthcare access.

### 4.6 Limitations

This study has several limitations. First, the availability and quality of raw data present constraints in estimating both the overall disease burden and risk-attributed SIDS burden. Historical disease registries from 32 years ago, particularly in low- and middle-income countries, were notably incomplete. Additionally, smaller-population countries in GBD framework often lack sufficient data, necessitating predictive variables and regional extrapolation that could affect accuracy. Critically, foundational data quality and access vary substantially across low-SDI countries. Across selected areas such as sub-Saharan Africa and South Asia, incomplete death registration and limited surveillance systems create uncertainty in true SIDS burden estimates. GBD modeling in these areas may underestimate actual prevalence, introducing reporting biases and model uncertainties. These limitations can significantly affect trend interpretations—especially when observing seemingly “stable” or “declining” burden patterns. Such trends require careful consideration of whether they reflect genuine epidemiological shifts or represent artificial flattening/concealment of true dynamics due to data quality constraints. Second, the GBD 2021 risk factor classification, limited to environmental/occupational risks, behavioral risks, and metabolic risks, fails to incorporate all SIDS-related determinants discussed earlier. This incomplete coverage may restrict the comprehensiveness of risk factor analyses. Third, the COVID-19 pandemic has disrupted global healthcare systems, delaying diagnoses, treatments, and care continuity for SIDS patients, which could worsen post-pandemic burden. However, as COVID-19 is categorized separately in GBD 2021, its direct and indirect impacts on SIDS remain insufficiently explored. Limited time since the pandemic's start also hinders trend analysis, creating a need for longitudinal research to understand these connections.

## 5 Conclusions

In conclusion, while the global burden of SIDS shows a gradual decline, persistent disparities across regions underscore ongoing challenges. The DALYs attributed to SIDS remain significant, and its post-COVID-19 trend warrants sustained surveillance. Mitigating modifiable risk factors, optimizing maternal healthcare services, and prioritizing comprehensive antenatal screening programs are pivotal strategies to alleviate the burden of SIDS. Therefore, we call for global collaborative action: policymakers should prioritize funding for community-based education programs while integrating comprehensive antenatal screening protocols and enhanced monitoring schemes into existing maternal healthcare services, both measures could significantly reduce SIDS incidence.

## Data Availability

Publicly available datasets were analyzed in this study. This data can be found here: the data utilized in these analyses are publicly accessible through the GBD Results Tool, available at https://vizhub.healthdata.org/gbd-results/.
