# Supplementary material for: Global, regional, and national burden of sudden infant death syndrome and the impact of COVID-19: a trend and health inequality analysis based on the global burden of disease study 2021
Source: Front Pediatr. 2025 Jul 25;13:1623238. doi: 10.3389/fped.2025.1623238 (PMC12331577; doi:10.3389/fped.2025.1623238)
Supplement: Supplementary file 1 [file Table1.docx]

[Table S1. Global, regional, and national DALYs number of SIDS in 1990 and 2019 2](#_Toc195120342)

[Figure S1. The ASDR trends of SIDS for global and five SDI regions. 13](#_Toc195120343)

[Table S2. The ASDR trends of SIDS for global and five SDI regions. 14](#_Toc195120344)

[Table S3. National ASDR of SIDS in 1990 and 2021, EAPCs of 1990-2021 and 2019-2021 19](#_Toc195120345)

[Figure S2. The proportion of DALYs attributable to risk factors of SIDS for 21 geographic regions in 2021. 29](#_Toc195120346)

[Table S4. The proportion of DALYs attributable to risk factors of SIDS for global, five SDI regions, and 21 geographic regions in 2021 30](#_Toc195120347)

[Table S5. ASDR of SIDS with frontier analysis across all countries and territories in 2021 33](#_Toc195120348)

Table S1. Global, regional, and national DALYs number of SIDS in 1990 and 2019

| Location | DALYs number (95% UI) |  |
| --- | --- | --- |
|  | 1990 | 2021 |
| Global | 6794659.76  (4121026.03 to 10289656.44) | 2746174.49  (1598180.42 to 3686289.79) |
| **Sex** |  |  |
| Female | 3399486.70  (1611240.58 to 5663274.55) | 1259805.70  (585867.02 to 1752413.44) |
| Male | 3395173.06  (1788727.10 to 5364023.52) | 1486368.80  (721571.21 to 2241382.43) |
| **SDI** |  |  |
| Low SDI | 1761821.05  (864011.55 to 2809316.20) | 1326431.33  (690406.38 to 1942045.37) |
| Low-middle SDI | 2672925.92  (1299197.46 to 4859910.59) | 838441.51  (460421.73 to 1239117.82) |
| Middle SDI | 989022.62  (583424.11 to 1416388.82) | 322450.57  (200345.06 to 446089.81) |
| High-middle SDI | 379694.61  (276271.19 to 535134.68) | 94943.09  (66952.84 to 123029.41) |
| High SDI | 987580.13  (953398.32 to 1020384.60) | 162001.17  (141386.97 to 183364.52) |
| **Region** |  |  |
| Andean Latin America | 25427.27  (12137.90 to 44571.95) | 5991.85  (3425.56 to 9774.79) |
| Australasia | 48727.34  (45673.82 to 51887.08) | 4245.98  (3164.88 to 5509.85) |
| Caribbean | 15815.70  (8439.70 to 30930.36) | 9537.28  (4204.69 to 18184.54) |
| Central Asia | 20637.38  (8036.33 to 30843.22) | 16414.04  (10492.25 to 25539.37) |
| Central Europe | 28440.07  (22894.42 to 34771.33) | 5615.67  (3899.74 to 7933.26) |
| Central Latin America | 56415.45  (49086.69 to 65707.31) | 48620.12  (35751.39 to 66227.66) |
| Central Sub-Saharan Africa | 106169.49  (36928.11 to 228840.78) | 77962.31  (33277.69 to 148928.45) |
| East Asia | 312006.98  (172446.36 to 490145.52) | 51899.15  (22550.07 to 86663.58) |
| Eastern Europe | 76102.93  (65036.26 to 90831.33) | 23538.09  (19622.19 to 30007.10) |
| Eastern Sub-Saharan Africa | 721306.80  (350457.45 to 1174021.18) | 413069.04  (222352.85 to 611013.28) |
| High-income Asia Pacific | 32445.37  (25092.41 to 42723.46) | 6497.93  (4601.51 to 8557.33) |
| High-income North America | 495525.41  (474931.01 to 514943.95) | 107205.78  (90868.58 to 123472.32) |
| North Africa and Middle East | 915563.21  (536521.35 to 1382917.57) | 363016.79  (208093.45 to 545949.08) |
| Oceania | 10171.16  (3561.11 to 19317.37) | 12573.56  (5804.45 to 21680.66) |
| South Asia | 2480553.02  (1071093.46 to 4772141.51) | 667250.48  (355049.10 to 1047731.12) |
| Southeast Asia | 423070.89  (181605.60 to 718708.61) | 141036.47  (61255.89 to 210893.64) |
| Southern Latin America | 49605.42  (36754.32 to 65978.79) | 10626.52  (7777.35 to 14370.07) |
| Southern Sub-Saharan Africa | 35250.95  (13587.98 to 78307.35) | 25747.13  (9660.41 to 61298.59) |
| Tropical Latin America | 17693.05  (14871.89 to 20788.71) | 10650.49  (8214.73 to 13490.37) |
| Western Europe | 384914.35  (368055.28 to 402251.87) | 36780.10  (30829.46 to 43856.47) |
| Western Sub-Saharan Africa | 538817.51  (269377.43 to 841467.24) | 707895.69  (346509.41 to 1066855.53) |
| **Nation** |  |  |
| Afghanistan | 69444.73  (33523.11 to 131750.84) | 80496.55  (39650.84 to 136428.93) |
| Albania | 2098.86  (613.09 to 5201.35) | 245.58  (105.33 to 476.44) |
| Algeria | 35121.70  (11921.71 to 82685.69) | 17050.48  (8745.17 to 27881.24) |
| American Samoa | 24.33  (9.50 to 43.93) | 6.48  (2.96 to 11.85) |
| Andorra | 14.75  (6.28 to 24.59) | 1.59  (0.44 to 3.11) |
| Angola | 33179.61  (14183.64 to 58399.15) | 28604.55  (12939.17 to 50883.36) |
| Antigua and Barbuda | 0.00  (0.00 to 0.00) | 0.00  (0.00 to 0.00) |
| Argentina | 40954.59  (28626.38 to 56945.07) | 8773.61  (6093.45 to 12352.72) |
| Armenia | 117.32  (53.30 to 191.50) | 42.13  (25.66 to 66.51) |
| Australia | 36431.67  (33590.25 to 39574.06) | 2272.50  (1576.10 to 3149.61) |
| Austria | 10483.46  (9459.94 to 11508.07) | 726.38  (557.46 to 927.18) |
| Azerbaijan | 1257.00  (373.37 to 3339.02) | 987.67  (422.59 to 2080.83) |
| Bahamas | 93.99  (67.71 to 124.91) | 50.33  (33.71 to 71.86) |
| Bahrain | 346.19  (176.07 to 610.05) | 187.29  (108.08 to 299.54) |
| Bangladesh | 460354.06  (179105.35 to 1097610.64) | 73719.13  (27676.89 to 155588.76) |
| Barbados | 48.37  (35.51 to 66.84) | 12.43  (8.24 to 18.69) |
| Belarus | 304.78  (191.89 to 454.08) | 107.24  (61.00 to 171.87) |
| Belgium | 16237.95  (14668.41 to 17927.89) | 1218.75  (855.56 to 1709.91) |
| Belize | 439.35  (321.57 to 614.03) | 97.00  (66.53 to 141.60) |
| Benin | 9725.27  (3114.88 to 23615.94) | 11023.79  (3333.31 to 28232.59) |
| Bermuda | 28.82  (20.72 to 38.74) | 4.31  (3.00 to 6.05) |
| Bhutan | 1396.77  (475.03 to 3446.78) | 207.78  (92.75 to 400.41) |
| Bolivia (Plurinational State of) | 7774.19  (3020.12 to 15913.38) | 2168.37  (1106.07 to 3879.04) |
| Bosnia and Herzegovina | 550.13  (182.00 to 1414.67) | 231.93  (58.13 to 521.41) |
| Botswana | 634.83  (216.19 to 1409.17) | 584.24  (183.87 to 1438.69) |
| Brazil | 16820.08  (13517.49 to 19774.20) | 10150.94  (7712.45 to 12914.19) |
| Brunei Darussalam | 73.79  (6.24 to 160.28) | 42.60  (4.15 to 110.57) |
| Bulgaria | 544.23  (403.21 to 729.76) | 194.87  (119.99 to 322.70) |
| Burkina Faso | 28452.66  (10827.71 to 55735.86) | 30025.32  (10364.44 to 75225.47) |
| Burundi | 16482.25  (5724.01 to 33358.30) | 7472.78  (2725.88 to 15453.21) |
| Cabo Verde | 655.76  (326.64 to 1145.41) | 105.94  (55.93 to 189.15) |
| Cambodia | 22506.28  (8680.24 to 45042.13) | 5136.27  (2069.33 to 10916.77) |
| Cameroon | 16474.04  (6243.46 to 37053.39) | 25763.94  (10101.25 to 54240.24) |
| Canada | 29193.13  (26004.95 to 32414.33) | 1855.61  (1266.64 to 2605.38) |
| Central African Republic | 6429.24  (1914.76 to 15852.35) | 6047.39  (2140.12 to 13683.51) |
| Chad | 23658.60  (10423.21 to 47807.72) | 46213.02  (20745.69 to 83493.67) |
| Chile | 7014.51  (5796.68 to 8485.62) | 1312.37  (969.49 to 1660.09) |
| China | 295849.40  (159278.78 to 474653.71) | 47742.43  (19837.99 to 81110.04) |
| Colombia | 7612.16  (6075.49 to 9319.82) | 4296.09  (2828.94 to 6321.31) |
| Comoros | 1249.90  (511.91 to 2614.77) | 372.58  (134.86 to 852.41) |
| Congo | 2544.59  (767.43 to 6678.61) | 1674.93  (645.43 to 3798.26) |
| Cook Islands | 7.54  (2.80 to 14.16) | 2.30  (0.43 to 4.81) |
| Costa Rica | 561.95  (427.55 to 726.72) | 190.11  (102.04 to 402.62) |
| Croatia | 1248.98  (981.14 to 1544.24) | 346.12  (229.46 to 563.71) |
| Cuba | 431.54  (340.72 to 548.24) | 45.96  (29.71 to 67.29) |
| Cyprus | 633.74  (196.21 to 1213.96) | 147.14  (63.59 to 281.52) |
| Czechia | 2266.58  (1809.87 to 2824.22) | 709.27  (459.24 to 1052.64) |
| Côte d’Ivoire | 28355.52  (11620.47 to 53825.72) | 21118.40  (8628.88 to 46345.85) |
| Democratic People's Republic of Korea | 5842.82  (2002.18 to 13920.50) | 2456.90  (1078.67 to 4895.53) |
| Democratic Republic of the Congo | 61671.34  (15684.86 to 156985.56) | 40493.86  (15073.91 to 86960.59) |
| Denmark | 1276.10  (1025.46 to 1569.66) | 152.34  (107.08 to 211.49) |
| Djibouti | 410.91  (99.69 to 1161.04) | 446.03  (143.69 to 1082.24) |
| Dominica | 8.76  (4.14 to 16.44) | 3.22  (1.35 to 6.88) |
| Dominican Republic | 1591.31  (573.13 to 3597.70) | 960.01  (432.82 to 1804.62) |
| Ecuador | 3836.85  (2627.58 to 5909.23) | 878.16  (559.18 to 1357.34) |
| Egypt | 174311.05  (84939.87 to 312908.32) | 53871.88  (27234.12 to 90044.36) |
| El Salvador | 1840.76  (755.18 to 3370.28) | 581.85  (222.64 to 1178.32) |
| Equatorial Guinea | 1559.96  (728.09 to 2854.78) | 650.85  (242.30 to 1396.66) |
| Eritrea | 10874.40  (4345.62 to 26591.60) | 7000.61  (3078.71 to 13252.10) |
| Estonia | 461.19  (324.50 to 654.53) | 58.88  (39.52 to 85.95) |
| Eswatini | 892.61  (282.15 to 1907.85) | 569.46  (146.35 to 1589.47) |
| Ethiopia | 319247.84  (126504.99 to 708720.19) | 116634.41  (56320.99 to 206189.41) |
| Fiji | 266.85  (91.23 to 537.97) | 164.21  (73.66 to 296.42) |
| Finland | 1722.43  (1433.12 to 2024.12) | 224.75  (170.31 to 297.17) |
| France | 105125.97  (97404.45 to 113105.31) | 10531.11  (8146.21 to 13777.70) |
| Gabon | 784.75  (295.70 to 1767.53) | 490.74  (201.24 to 971.81) |
| Gambia | 1593.24  (777.18 to 2839.73) | 1121.17  (571.98 to 1911.80) |
| Georgia | 1.02  (0.62 to 1.58) | 328.35  (210.83 to 486.85) |
| Germany | 96474.81  (88501.43 to 104715.87) | 7881.72  (5880.85 to 10419.74) |
| Ghana | 23763.41  (11253.80 to 41031.52) | 15065.42  (6375.04 to 29127.14) |
| Greece | 1012.29  (823.69 to 1217.97) | 173.12  (121.84 to 245.01) |
| Greenland | 143.98  (57.08 to 279.76) | 33.69  (18.26 to 55.07) |
| Grenada | 20.45  (14.63 to 28.38) | 0.03  (0.02 to 0.04) |
| Guam | 57.24  (22.70 to 101.38) | 26.59  (13.32 to 47.73) |
| Guatemala | 8423.44  (6406.41 to 11581.53) | 6187.92  (4578.79 to 8432.84) |
| Guinea | 25828.88  (11768.22 to 44358.52) | 20308.56  (8812.55 to 38211.88) |
| Guinea-Bissau | 3518.63  (1579.86 to 6148.70) | 2399.73  (1079.96 to 4344.87) |
| Guyana | 283.42  (177.58 to 409.70) | 67.68  (40.70 to 103.38) |
| Haiti | 10559.44  (4238.57 to 25470.28) | 7750.45  (3054.30 to 16172.05) |
| Honduras | 2900.31  (1144.47 to 5840.98) | 1287.28  (351.58 to 2987.07) |
| Hungary | 2857.24  (2282.68 to 3431.66) | 1035.33  (703.55 to 1557.81) |
| Iceland | 281.81  (239.99 to 325.03) | 30.47  (23.20 to 39.52) |
| India | 1383519.07  (645646.93 to 2532935.17) | 381394.89  (187003.25 to 628218.34) |
| Indonesia | 199819.32  (80511.21 to 370907.12) | 55428.23  (23981.01 to 90205.50) |
| Iran (Islamic Republic of) | 101737.85  (51684.03 to 184122.55) | 6562.71  (3648.04 to 11014.51) |
| Iraq | 46004.54  (23581.08 to 77758.30) | 22342.58  (11158.00 to 37647.66) |
| Ireland | 6382.31  (5787.89 to 7009.47) | 841.33  (588.77 to 1173.67) |
| Israel | 3818.38  (2537.67 to 4986.23) | 753.34  (540.55 to 1013.98) |
| Italy | 6696.69  (5920.02 to 7490.54) | 1033.46  (764.86 to 1361.58) |
| Jamaica | 375.10  (285.07 to 493.01) | 61.36  (37.88 to 96.66) |
| Japan | 25618.22  (23466.67 to 27997.75) | 4988.10  (4072.81 to 6093.26) |
| Jordan | 3947.35  (1907.63 to 6914.27) | 3108.07  (1746.84 to 5084.19) |
| Kazakhstan | 8074.88  (2577.50 to 12333.20) | 4303.87  (2632.01 to 6878.55) |
| Kenya | 42051.27  (21834.48 to 67776.05) | 20263.23  (11319.48 to 33753.23) |
| Kiribati | 98.34  (30.33 to 237.09) | 44.73  (17.65 to 79.81) |
| Kuwait | 873.91  (619.32 to 1147.71) | 1023.17  (755.27 to 1356.80) |
| Kyrgyzstan | 1653.73  (1044.13 to 2587.55) | 590.38  (291.64 to 1150.11) |
| Lao People's Democratic Republic | 10617.53  (3773.78 to 22362.87) | 4168.73  (1581.72 to 8083.44) |
| Latvia | 1823.11  (1352.70 to 2394.43) | 297.14  (213.69 to 401.08) |
| Lebanon | 2031.87  (836.15 to 3889.78) | 1037.49  (504.62 to 1985.77) |
| Lesotho | 1896.87  (887.10 to 3512.97) | 725.91  (181.12 to 2232.97) |
| Liberia | 9633.48  (3734.28 to 20502.61) | 4319.97  (1822.91 to 8752.48) |
| Libya | 3291.08  (1305.95 to 7317.33) | 1272.37  (570.91 to 2290.44) |
| Lithuania | 595.80  (424.60 to 825.31) | 182.87  (132.65 to 247.65) |
| Luxembourg | 722.68  (651.11 to 801.54) | 94.54  (70.56 to 126.54) |
| Madagascar | 25540.49  (9970.11 to 58717.68) | 27174.13  (12274.78 to 47981.52) |
| Malawi | 27479.01  (8220.06 to 71726.51) | 9753.87  (3462.98 to 22629.79) |
| Malaysia | 4565.98  (1871.85 to 8972.36) | 2636.08  (918.53 to 5256.49) |
| Maldives | 150.25  (66.55 to 274.29) | 40.20  (16.25 to 74.51) |
| Mali | 28088.22  (10669.27 to 48790.90) | 27149.15  (9329.34 to 61158.53) |
| Malta | 40.47  (31.77 to 51.81) | 5.88  (4.14 to 7.98) |
| Marshall Islands | 33.01  (12.97 to 62.45) | 12.17  (5.61 to 22.41) |
| Mauritania | 3089.54  (1277.56 to 5618.79) | 2219.57  (991.28 to 4139.64) |
| Mauritius | 197.56  (144.32 to 264.73) | 175.52  (120.00 to 248.85) |
| Mexico | 31463.41  (27640.02 to 36384.66) | 33022.81  (23650.50 to 46040.73) |
| Micronesia (Federated States of) | 93.36  (37.57 to 169.54) | 18.56  (9.44 to 33.39) |
| Monaco | 10.48  (3.78 to 19.10) | 7.81  (2.15 to 13.30) |
| Mongolia | 474.58  (122.09 to 1279.66) | 516.46  (191.13 to 1161.54) |
| Montenegro | 171.50  (70.27 to 371.57) | 42.14  (9.41 to 100.11) |
| Morocco | 109516.63  (55609.66 to 219887.07) | 17758.96  (8336.59 to 31080.28) |
| Mozambique | 59554.41  (22596.43 to 111330.34) | 34269.18  (12825.43 to 74230.29) |
| Myanmar | 57940.07  (22337.64 to 109050.94) | 25986.57  (10411.08 to 47789.70) |
| Namibia | 971.85  (385.47 to 1878.85) | 810.09  (259.48 to 1761.33) |
| Nauru | 6.28  (2.07 to 13.17) | 3.59  (1.47 to 6.82) |
| Nepal | 71128.82  (26708.79 to 162848.91) | 13613.88  (6721.21 to 25309.45) |
| Netherlands | 10776.19  (9629.50 to 12010.57) | 1181.66  (890.58 to 1526.45) |
| New Zealand | 12295.67  (11274.52 to 13316.68) | 1973.48  (1529.26 to 2459.66) |
| Nicaragua | 2175.70  (1080.49 to 3862.10) | 855.20  (248.08 to 1653.53) |
| Niger | 42018.08  (18231.74 to 81380.41) | 50951.07  (20800.74 to 99424.55) |
| Nigeria | 255315.35  (121066.40 to 453292.33) | 429317.45  (186071.91 to 728042.61) |
| Niue | 0.87  (0.34 to 1.62) | 0.95  (0.51 to 1.52) |
| North Macedonia | 927.80  (229.08 to 2285.96) | 128.97  (50.97 to 257.71) |
| Northern Mariana Islands | 10.21  (3.76 to 19.59) | 2.78  (1.35 to 5.10) |
| Norway | 6376.78  (5848.91 to 6918.83) | 482.57  (404.98 to 575.40) |
| Oman | 2396.14  (1265.60 to 4011.70) | 1230.01  (645.77 to 1970.54) |
| Pakistan | 564154.28  (195671.27 to 1157154.06) | 198314.80  (89410.51 to 371961.65) |
| Palau | 6.12  (1.92 to 14.80) | 2.01  (0.97 to 3.56) |
| Palestine | 2148.98  (745.34 to 4523.77) | 2013.39  (1047.46 to 3371.63) |
| Panama | 136.70  (106.95 to 176.14) | 441.26  (308.42 to 604.29) |
| Papua New Guinea | 8203.07  (2827.13 to 15854.19) | 11270.49  (5263.97 to 19672.25) |
| Paraguay | 872.97  (167.47 to 2014.23) | 499.55  (134.22 to 986.85) |
| Peru | 13816.23  (5970.29 to 27351.54) | 2945.32  (1386.94 to 5469.63) |
| Philippines | 58779.83  (19974.70 to 104544.35) | 25450.84  (11039.48 to 42513.32) |
| Poland | 12399.54  (10166.23 to 15004.00) | 1304.15  (860.71 to 1916.94) |
| Portugal | 1114.70  (887.09 to 1373.67) | 83.39  (59.61 to 113.90) |
| Puerto Rico | 504.90  (381.91 to 651.58) | 0.04  (0.03 to 0.06) |
| Qatar | 236.18  (93.63 to 433.99) | 297.53  (133.92 to 575.91) |
| Republic of Korea | 6699.96  (537.56 to 16753.95) | 1457.41  (199.86 to 3252.44) |
| Republic of Moldova | 275.58  (183.01 to 392.52) | 327.31  (212.54 to 488.69) |
| Romania | 1025.26  (784.64 to 1363.48) | 248.41  (156.54 to 376.94) |
| Russian Federation | 51514.20  (45219.55 to 58931.59) | 17633.04  (14828.04 to 21255.67) |
| Rwanda | 23997.42  (10595.19 to 43070.61) | 5896.57  (2211.72 to 12435.13) |
| Saint Kitts and Nevis | 14.13  (10.28 to 18.89) | 2.50  (1.60 to 3.92) |
| Saint Lucia | 115.64  (87.87 to 148.60) | 19.31  (12.28 to 29.47) |
| Saint Vincent and the Grenadines | 40.91  (29.22 to 55.02) | 2.59  (1.65 to 3.90) |
| Samoa | 137.63  (54.66 to 247.43) | 69.56  (28.69 to 149.89) |
| San Marino | 8.60  (3.03 to 15.04) | 1.96  (0.75 to 3.48) |
| Sao Tome and Principe | 209.01  (94.59 to 413.12) | 63.92  (28.91 to 115.53) |
| Saudi Arabia | 17896.36  (7848.62 to 33453.13) | 4036.23  (1510.65 to 7657.81) |
| Senegal | 16250.40  (6844.08 to 29214.03) | 9582.02  (3652.06 to 20764.85) |
| Serbia | 2531.71  (1013.71 to 4573.79) | 433.64  (117.80 to 872.74) |
| Seychelles | 11.52  (4.37 to 20.59) | 10.67  (4.60 to 20.21) |
| Sierra Leone | 16437.77  (6030.67 to 35951.93) | 7053.45  (1804.91 to 19703.31) |
| Singapore | 53.41  (18.30 to 96.16) | 9.82  (6.13 to 14.97) |
| Slovakia | 1007.52  (419.04 to 1772.16) | 548.09  (213.66 to 1028.83) |
| Slovenia | 355.92  (262.06 to 473.64) | 65.43  (43.04 to 99.68) |
| Solomon Islands | 378.57  (133.83 to 730.39) | 255.61  (118.58 to 479.83) |
| Somalia | 29183.65  (13779.66 to 53794.92) | 45848.36  (22568.13 to 79989.78) |
| South Africa | 22382.71  (7323.24 to 54806.29) | 14144.16  (4880.90 to 36697.50) |
| South Sudan | 26753.81  (11076.97 to 48747.95) | 34481.99  (16012.70 to 63557.46) |
| Spain | 11720.36  (10054.68 to 13589.00) | 2160.05  (1563.25 to 2980.63) |
| Sri Lanka | 2336.24  (994.95 to 4174.84) | 1465.04  (471.29 to 2827.21) |
| Sudan | 108194.03  (52492.50 to 216855.45) | 60860.94  (29290.49 to 127906.70) |
| Suriname | 80.40  (29.68 to 168.91) | 36.60  (17.12 to 69.25) |
| Sweden | 9616.15  (8838.58 to 10441.56) | 936.24  (765.07 to 1167.71) |
| Switzerland | 1866.06  (1516.05 to 2271.40) | 239.45  (164.36 to 351.77) |
| Syrian Arab Republic | 36260.97  (18731.91 to 64958.91) | 2648.07  (903.27 to 5105.20) |
| Taiwan (Province of China) | 10314.76  (8805.63 to 11961.63) | 1699.82  (1198.68 to 2327.99) |
| Tajikistan | 1486.96  (520.62 to 3452.53) | 2867.97  (1113.78 to 6908.99) |
| Thailand | 13675.74  (6002.04 to 23560.27) | 3504.47  (1565.90 to 6203.80) |
| Timor-Leste | 1886.50  (741.35 to 4681.16) | 977.51  (411.71 to 1700.89) |
| Togo | 5731.88  (2157.96 to 10951.43) | 4085.53  (1642.67 to 7935.43) |
| Tokelau | 0.77  (0.32 to 1.40) | 1.38  (0.61 to 2.51) |
| Tonga | 55.10  (20.39 to 98.84) | 24.90  (11.04 to 49.87) |
| Trinidad and Tobago | 632.19  (469.48 to 839.44) | 99.04  (67.56 to 142.12) |
| Tunisia | 11366.71  (4758.25 to 22717.99) | 2519.32  (1330.32 to 4326.15) |
| Turkmenistan | 1309.20  (336.58 to 2034.23) | 949.36  (527.76 to 1572.84) |
| Tuvalu | 10.65  (2.14 to 29.53) | 2.85  (1.32 to 5.35) |
| Turkey | 100534.69  (35883.56 to 200057.29) | 16088.32  (8696.69 to 26114.00) |
| Uganda | 47111.89  (19207.90 to 87443.16) | 32334.50  (12881.79 to 66459.56) |
| Ukraine | 21128.28  (14000.54 to 31202.25) | 4931.60  (3240.48 to 8574.13) |
| United Arab Emirates | 989.94  (510.73 to 1745.10) | 645.43  (315.96 to 1160.23) |
| United Kingdom | 92184.85  (88429.32 to 96620.04) | 7838.68  (6439.69 to 9155.36) |
| United Republic of Tanzania | 70784.53  (21741.26 to 168673.37) | 59426.95  (22922.28 to 117703.22) |
| United States of America | 466176.93  (446414.11 to 484957.34) | 105314.81  (89369.39 to 121542.28) |
| United States Virgin Islands | 11.45  (5.26 to 20.78) | 1.66  (0.46 to 3.57) |
| Uruguay | 1633.96  (1317.20 to 2013.11) | 539.95  (377.15 to 747.87) |
| Uzbekistan | 6262.69  (1996.85 to 9583.29) | 5827.85  (3208.01 to 9314.13) |
| Vanuatu | 132.37  (52.33 to 251.74) | 99.91  (47.57 to 189.48) |
| Venezuela (Bolivarian Republic of) | 1301.01  (1043.83 to 1595.51) | 1757.59  (1157.53 to 2713.15) |
| Viet Nam | 49972.06  (19104.98 to 87996.03) | 15859.63  (5866.81 to 31540.79) |
| Yemen | 88411.46  (43464.71 to 153971.96) | 67627.41  (30389.93 to 136349.23) |
| Zambia | 20069.53  (6675.04 to 45967.79) | 11334.24  (4038.58 to 24878.81) |
| Zimbabwe | 8472.07  (3472.17 to 18203.74) | 8913.27  (3231.51 to 21420.24) |

DALYs = disability-adjusted life years, SIDS= sudden infant death syndrome, UI = uncertainty intervals, SDI= socio-demographic index





Figure S1. The ASDR trends of SIDS for global and five SDI regions.

ASDR= Age standardized disability-adjusted life years rate, SIDS= sudden infant death syndrome, SDI= socio-demographic index

Table S2. The ASDR trends of SIDS for global and five SDI regions.

| Location | Year | ASDR | 95% LUI | 95% UUI |
| --- | --- | --- | --- | --- |
| Global | 1990 | 107.64 | 65.31 | 162.96 |
|  | 1991 | 106.78 | 63.96 | 162.83 |
|  | 1992 | 105.72 | 62.56 | 162.16 |
|  | 1993 | 104.5 | 61.41 | 159.15 |
|  | 1994 | 102.53 | 60.12 | 154.27 |
|  | 1995 | 100.18 | 57.66 | 150.72 |
|  | 1996 | 97.74 | 56.35 | 146.47 |
|  | 1997 | 95.78 | 55.06 | 142.81 |
|  | 1998 | 93.65 | 54.21 | 140.74 |
|  | 1999 | 91.5 | 52.77 | 139.53 |
|  | 2000 | 89.78 | 51.73 | 138.28 |
|  | 2001 | 87.92 | 50.06 | 136.19 |
|  | 2002 | 86.15 | 48.96 | 132.2 |
|  | 2003 | 84.27 | 48.53 | 128.23 |
|  | 2004 | 82.29 | 46.93 | 124.29 |
|  | 2005 | 80.23 | 45.5 | 122.08 |
|  | 2006 | 78.33 | 44.52 | 119.81 |
|  | 2007 | 75.44 | 43.46 | 113.73 |
|  | 2008 | 71.17 | 42.2 | 104.6 |
|  | 2009 | 66.65 | 40.66 | 94.62 |
|  | 2010 | 63.35 | 38.27 | 88.25 |
|  | 2011 | 61.17 | 36.99 | 83.83 |
|  | 2012 | 59.02 | 36.06 | 80.7 |
|  | 2013 | 57.19 | 34.49 | 77.17 |
|  | 2014 | 55.42 | 33.24 | 74.35 |
|  | 2015 | 53.47 | 31.69 | 71.8 |
|  | 2016 | 51.75 | 30.7 | 69.23 |
|  | 2017 | 50.51 | 29.98 | 67.73 |
|  | 2018 | 49.68 | 29.18 | 66.34 |
|  | 2019 | 49.19 | 28.95 | 65.66 |
|  | 2020 | 45.19 | 26.32 | 61.4 |
|  | 2021 | 44.16 | 25.7 | 59.26 |
| Low SDI | 1990 | 172.81 | 84.81 | 275.36 |
|  | 1991 | 172.01 | 82.88 | 275.15 |
|  | 1992 | 171.4 | 83.23 | 276.07 |
|  | 1993 | 170.63 | 84.3 | 277.18 |
|  | 1995 | 168.34 | 83.42 | 270.25 |
|  | 1994 | 170.17 | 82.72 | 278.47 |
|  | 1996 | 165.55 | 81.6 | 268.12 |
|  | 1997 | 162.59 | 79.79 | 263.57 |
|  | 1998 | 159.73 | 77.91 | 264.18 |
|  | 1999 | 157.1 | 77.85 | 261.87 |
|  | 2000 | 155.02 | 75.97 | 259.27 |
|  | 2001 | 152.34 | 73.88 | 254.2 |
|  | 2002 | 149.59 | 71.89 | 252.96 |
|  | 2003 | 146.78 | 71.16 | 247.83 |
|  | 2004 | 143.95 | 70.4 | 243.26 |
|  | 2005 | 140.72 | 68.34 | 237.04 |
|  | 2006 | 136.81 | 66.96 | 226.63 |
|  | 2007 | 131.65 | 65.78 | 214.83 |
|  | 2008 | 125.68 | 63.94 | 202.46 |
|  | 2009 | 119.06 | 61.21 | 187.6 |
|  | 2010 | 113.38 | 59.66 | 176.85 |
|  | 2011 | 109.56 | 58.63 | 171.14 |
|  | 2012 | 106.65 | 56.3 | 165.31 |
|  | 2013 | 104.04 | 55.19 | 157.54 |
|  | 2014 | 101.46 | 53.83 | 149.16 |
|  | 2015 | 98.13 | 51.75 | 140.06 |
|  | 2016 | 95.43 | 51.5 | 135.32 |
|  | 2017 | 92.59 | 49.14 | 132.75 |
|  | 2018 | 89.76 | 47.57 | 129.5 |
|  | 2019 | 86.96 | 46.05 | 126.97 |
|  | 2020 | 80.37 | 42.32 | 118.12 |
|  | 2021 | 77.75 | 40.48 | 113.81 |
| Low-middle SDI | 1990 | 147.96 | 71.99 | 268.96 |
|  | 1991 | 146.85 | 71.1 | 263.09 |
|  | 1992 | 146.04 | 70.43 | 260.07 |
|  | 1993 | 143.8 | 69.34 | 257.43 |
|  | 1994 | 139.57 | 68.62 | 243.83 |
|  | 1995 | 135.42 | 66.55 | 230.01 |
|  | 1996 | 130.6 | 64.97 | 220.2 |
|  | 1997 | 126.82 | 63.88 | 214.83 |
|  | 1998 | 122.8 | 62.32 | 206.23 |
|  | 1999 | 119.15 | 59.82 | 203.18 |
|  | 2000 | 116.59 | 59.43 | 197.95 |
|  | 2001 | 114.39 | 57.93 | 197.54 |
|  | 2002 | 111.46 | 57.02 | 189.01 |
|  | 2003 | 107.64 | 56.35 | 179.17 |
|  | 2004 | 104.27 | 55.01 | 173.49 |
|  | 2005 | 101.33 | 53.96 | 165.13 |
|  | 2006 | 99.14 | 51.84 | 159.95 |
|  | 2007 | 94.97 | 49.69 | 151.19 |
|  | 2008 | 87.51 | 47.44 | 136.18 |
|  | 2009 | 79.24 | 46.29 | 115.58 |
|  | 2010 | 73.14 | 42.88 | 102.9 |
|  | 2011 | 69.31 | 39.62 | 96.6 |
|  | 2012 | 66.44 | 37.31 | 93.31 |
|  | 2013 | 64.35 | 35.6 | 92.4 |
|  | 2014 | 62.12 | 33.46 | 89.8 |
|  | 2015 | 60.31 | 32.24 | 89.79 |
|  | 2016 | 58.8 | 31.27 | 88.02 |
|  | 2017 | 57.15 | 30.52 | 85.33 |
|  | 2018 | 55.44 | 29.69 | 82.39 |
|  | 2019 | 54.44 | 29.83 | 80.8 |
|  | 2020 | 46.72 | 25.53 | 70 |
|  | 2021 | 44.92 | 24.67 | 66.38 |
| Middle SDI | 1990 | 49.7 | 29.32 | 71.19 |
|  | 1991 | 48.41 | 28.03 | 68.11 |
|  | 1992 | 47.6 | 27.34 | 65.88 |
|  | 1993 | 46.73 | 27.07 | 63.64 |
|  | 1994 | 45.71 | 26.65 | 62.66 |
|  | 1995 | 44.69 | 26.02 | 60.98 |
|  | 1996 | 43.37 | 25.06 | 59.14 |
|  | 1997 | 42.11 | 24.36 | 57.15 |
|  | 1998 | 40.67 | 23.9 | 55.67 |
|  | 1999 | 39.11 | 23.62 | 53.89 |
|  | 2000 | 37.79 | 23.13 | 51.81 |
|  | 2002 | 35.1 | 21.41 | 47.68 |
|  | 2001 | 36.31 | 22.2 | 49.73 |
|  | 2003 | 34.35 | 21.22 | 46.91 |
|  | 2004 | 33.5 | 20.92 | 45.47 |
|  | 2005 | 32.54 | 20.29 | 44.41 |
|  | 2006 | 32.09 | 19.43 | 43.39 |
|  | 2007 | 31.17 | 18.93 | 42.41 |
|  | 2008 | 29.89 | 17.96 | 40.87 |
|  | 2009 | 28.83 | 17.75 | 39.43 |
|  | 2010 | 28.33 | 17.22 | 38.58 |
|  | 2011 | 27.96 | 16.95 | 38.87 |
|  | 2012 | 27.18 | 16.03 | 37.48 |
|  | 2013 | 26.66 | 15.92 | 36.4 |
|  | 2014 | 26.14 | 15.37 | 35.53 |
|  | 2015 | 25.38 | 15.32 | 34.36 |
|  | 2016 | 24.29 | 14.94 | 32.89 |
|  | 2017 | 23.86 | 14.75 | 32.1 |
|  | 2018 | 23.67 | 14.69 | 32.28 |
|  | 2019 | 23.47 | 14.73 | 31.74 |
|  | 2020 | 21.43 | 13.38 | 28.99 |
|  | 2021 | 20.7 | 12.87 | 28.66 |
| High-middle SDI | 1990 | 42.89 | 31.21 | 60.44 |
|  | 1991 | 42.24 | 30.76 | 58.89 |
|  | 1992 | 41.31 | 30.02 | 57.63 |
|  | 1993 | 40.41 | 29.58 | 58 |
|  | 1994 | 39.62 | 28.8 | 56.96 |
|  | 1995 | 38.83 | 28.29 | 55.79 |
|  | 1996 | 37.85 | 27.81 | 53.76 |
|  | 1997 | 37.13 | 27.52 | 51.91 |
|  | 1998 | 36.44 | 27.37 | 50.88 |
|  | 1999 | 35.26 | 26.91 | 48.28 |
|  | 2000 | 34.16 | 26.31 | 45.49 |
|  | 2001 | 33.25 | 26.02 | 43.58 |
|  | 2002 | 32.71 | 25.98 | 41.26 |
|  | 2003 | 31.98 | 25.87 | 40.28 |
|  | 2004 | 30.36 | 24.75 | 37.75 |
|  | 2005 | 28.39 | 23.54 | 34.56 |
|  | 2006 | 26.97 | 22.49 | 32.73 |
|  | 2007 | 25.97 | 21.62 | 31.23 |
|  | 2008 | 24.84 | 20.6 | 29.84 |
|  | 2009 | 23.89 | 19.57 | 28.68 |
|  | 2010 | 23.25 | 19.03 | 27.61 |
|  | 2011 | 22.95 | 18.9 | 26.72 |
|  | 2012 | 22.41 | 18.6 | 26.42 |
|  | 2013 | 21.09 | 17.38 | 24.87 |
|  | 2014 | 19.86 | 16.12 | 23.77 |
|  | 2015 | 18.25 | 14.39 | 22.27 |
|  | 2016 | 17.11 | 13.13 | 20.91 |
|  | 2017 | 16.37 | 12.38 | 20.41 |
|  | 2018 | 15.98 | 12 | 20.28 |
|  | 2019 | 15.9 | 11.9 | 20.17 |
|  | 2020 | 16.67 | 12 | 21.58 |
|  | 2021 | 16.42 | 11.58 | 21.27 |
| High SDI | 1990 | 162.8 | 157.16 | 168.21 |
|  | 1991 | 155.83 | 150.7 | 161.22 |
|  | 1992 | 143.33 | 138.61 | 148.48 |
|  | 1993 | 134.19 | 129.62 | 138.86 |
|  | 1994 | 123.33 | 119.23 | 127.6 |
|  | 1995 | 110.77 | 106.9 | 114.63 |
|  | 1996 | 101.96 | 97.91 | 105.68 |
|  | 1997 | 95.37 | 91.74 | 99.03 |
|  | 1998 | 89.53 | 86.37 | 93.05 |
|  | 1999 | 84.03 | 80.78 | 87.19 |
|  | 2000 | 79.16 | 76.01 | 82.19 |
|  | 2001 | 74.39 | 71.41 | 77.44 |
|  | 2002 | 71.17 | 68.24 | 74.31 |
|  | 2003 | 68.62 | 65.53 | 71.67 |
|  | 2004 | 66.16 | 63.44 | 68.82 |
|  | 2005 | 64.96 | 62.24 | 67.52 |
|  | 2006 | 63.68 | 61.03 | 66.16 |
|  | 2007 | 62.74 | 59.92 | 65.09 |
|  | 2008 | 60.15 | 57.62 | 62.27 |
|  | 2009 | 56.75 | 54.31 | 58.91 |
|  | 2010 | 52.96 | 50.68 | 54.97 |
|  | 2011 | 49.94 | 47.58 | 52.15 |
|  | 2012 | 47.02 | 44.78 | 49.39 |
|  | 2013 | 44.37 | 41.98 | 46.78 |
|  | 2014 | 41.88 | 39.64 | 44.14 |
|  | 2015 | 40.56 | 38.39 | 42.83 |
|  | 2016 | 38.66 | 36.7 | 40.74 |
|  | 2017 | 36.55 | 34.38 | 38.73 |
|  | 2018 | 35.69 | 33.41 | 37.96 |
|  | 2019 | 35.07 | 32.6 | 37.76 |
|  | 2020 | 33.26 | 29.92 | 36.73 |
|  | 2021 | 32.22 | 28.12 | 36.47 |

ASDR= Age standardized disability-adjusted life years rate, SIDS= sudden infant death syndrome, SDI= socio-demographic index, LUI= lower uncertainty intervals. UUI= upper uncertainty intervals

Table S3. National ASDR of SIDS in 1990 and 2021, EAPCs of 1990-2021 and 2019-2021

| Location | ASDR per 100,000 population (95% UI) | | EAPC of ASDR (95% CI) | |
| --- | --- | --- | --- | --- |
|  | 1990 | 2021 | 1990-2021 | 2019-2021 |
| Afghanistan | 346.09  (166.96 to 655.34) | 139.98  (68.96 to 237.06) | -3.30  (-3.91 to -2.69) | -2.77  (-5.13 to -0.36) |
| Albania | 53.78  (15.71 to 133.28) | 18.07  (7.76 to 35.05) | -4.12  (-4.35 to -3.89) | -4.64  (-14.98 to 6.97) |
| Algeria | 95.86  (32.53 to 225.72) | 38.51  (19.75 to 62.96) | -2.61  (-2.70 to -2.53) | -4.38  (-13.32 to 5.49) |
| American Samoa | 28.88  (11.27 to 52.15) | 19.85  (9.08 to 36.30) | -1.40  (-1.64 to -1.16) | -1.61  (-44.87 to 75.60) |
| Andorra | 58.38  (24.85 to 97.33) | 7.08  (1.95 to 13.82) | -5.11  (-5.57 to -4.65) | -29.39  (-90.83 to 443.58) |
| Angola | 144.70  (61.60 to 255.08) | 49.90  (22.56 to 88.78) | -3.19  (-3.41 to -2.97) | -6.73  (-17.26 to 5.14) |
| Antigua and Barbuda | 0.00  (0.00 to 0.00) | 0.00  (0.00 to 0.00) | -1.20  (-1.65 to -0.75) | -5.11  (-6.13 to -4.08) |
| Argentina | 123.07  (86.02 to 171.12) | 33.70  (23.41 to 47.45) | -3.91  (-4.30 to -3.52) | -8.47  (-45.84 to 54.66) |
| Armenia | 3.19  (1.45 to 5.22) | 2.48  (1.51 to 3.92) | 0.15  (-1.05 to 1.36) | -3.91  (-8.93 to 1.39) |
| Australia | 293.16  (270.30 to 318.44) | 15.78  (10.94 to 21.86) | -8.01  (-8.48 to -7.54) | -12.35  (-46.56 to 43.75) |
| Austria | 234.64  (211.74 to 257.56) | 17.51  (13.44 to 22.35) | -7.77  (-8.17 to -7.38) | -9.41  (-30.39 to 17.90) |
| Azerbaijan | 14.30  (4.24 to 37.98) | 15.15  (6.49 to 31.89) | -0.11  (-0.41 to 0.19) | -1.95  (-5.52 to 1.76) |
| Bahamas | 35.90  (25.85 to 47.72) | 25.90  (17.35 to 36.99) | -1.71  (-2.27 to -1.14) | -2.73  (-15.23 to 11.61) |
| Bahrain | 54.26  (27.60 to 95.61) | 21.71  (12.52 to 34.71) | -2.83  (-2.99 to -2.67) | -4.01  (-21.53 to 17.42) |
| Bangladesh | 234.67  (91.32 to 559.98) | 54.40  (20.43 to 114.80) | -4.77  (-5.19 to -4.33) | -7.16  (-7.51 to -6.82) |
| Barbados | 24.16  (17.74 to 33.38) | 9.73  (6.45 to 14.62) | -3.28  (-4.19 to -2.36) | -1.15  (-5.04 to 2.91) |
| Belarus | 4.34  (2.73 to 6.47) | 2.62  (1.49 to 4.20) | -2.39  (-2.91 to -1.87) | -7.27  (-19.93 to 7.39) |
| Belgium | 268.20  (242.28 to 296.14) | 21.81  (15.31 to 30.60) | -8.07  (-8.41 to -7.72) | -6.53  (-33.99 to 32.35) |
| Belize | 145.91  (106.81 to 203.91) | 26.20  (17.97 to 38.25) | -5.67  (-6.44 to -4.89) | -2.77  (-4.02 to -1.49) |
| Benin | 88.84  (28.34 to 216.82) | 44.67  (13.49 to 114.54) | -2.11  (-2.43 to -1.79) | -3.60  (-14.83 to 9.11) |
| Bermuda | 66.81  (48.03 to 89.83) | 17.96  (12.52 to 25.24) | -4.25  (-4.85 to -3.64) | -0.79  (-13.89 to 14.29) |
| Bhutan | 135.44  (46.01 to 334.31) | 34.62  (15.45 to 66.72) | -5.01  (-5.39 to -4.62) | -2.72  (-3.25 to -2.19) |
| Bolivia (Plurinational State of) | 72.64  (28.23 to 148.63) | 18.45  (9.41 to 33.00) | -4.51  (-4.64 to -4.39) | -4.13  (-5.26 to -3.00) |
| Bosnia and Herzegovina | 16.73  (5.54 to 43.00) | 17.04  (4.27 to 38.29) | -0.84  (-1.49 to -0.19) | -6.65  (-8.66 to -4.59) |
| Botswana | 28.85  (9.82 to 64.06) | 25.10  (7.90 to 61.82) | -0.47  (-0.52 to -0.42) | 0.03  (-1.69 to 1.77) |
| Brazil | 10.89  (8.75 to 12.80) | 6.30  (4.78 to 8.01) | -1.70  (-2.36 to -1.02) | -10.22  (-46.03 to 49.37) |
| Brunei Darussalam | 21.74  (1.84 to 47.21) | 14.28  (1.39 to 37.06) | -1.35  (-1.51 to -1.19) | -3.38  (-5.18 to -1.54) |
| Bulgaria | 11.02  (8.15 to 14.79) | 6.83  (4.20 to 11.31) | -2.14  (-2.74 to -1.53) | -2.84  (-18.43 to 15.71) |
| Burkina Faso | 133.83  (50.83 to 262.40) | 67.30  (23.21 to 168.76) | -3.39  (-3.92 to -2.85) | -2.47  (-5.16 to 0.30) |
| Burundi | 136.98  (47.56 to 277.19) | 33.69  (12.28 to 69.71) | -5.09  (-5.89 to -4.28) | -3.08  (-11.47 to 6.12) |
| Cabo Verde | 109.40  (54.45 to 191.10) | 25.62  (13.51 to 45.76) | -4.80  (-5.16 to -4.44) | -5.67  (-15.84 to 5.73) |
| Cambodia | 113.10  (43.65 to 226.84) | 29.92  (12.06 to 63.58) | -5.86  (-6.56 to -5.15) | 0.57  (-0.99 to 2.15) |
| Cameroon | 74.11  (28.02 to 166.89) | 52.25  (20.47 to 110.08) | -0.91  (-1.20 to -0.61) | -7.34  (-7.93 to -6.74) |
| Canada | 149.05  (132.78 to 165.49) | 10.33  (7.05 to 14.51) | -8.18  (-8.75 to -7.61) | -6.54  (-34.61 to 33.59) |
| Central African Republic | 111.29  (33.04 to 275.04) | 68.19  (24.10 to 154.33) | -1.62  (-1.84 to -1.40) | -3.37  (-4.66 to -2.06) |
| Chad | 161.77  (71.25 to 327.34) | 115.16  (51.59 to 207.66) | -1.14  (-1.23 to -1.05) | -2.28  (-4.05 to -0.47) |
| Chile | 47.69  (39.41 to 57.69) | 13.26  (9.79 to 16.77) | -3.72  (-5.43 to -1.98) | -16.49  (-65.54 to 102.38) |
| China | 26.80  (14.43 to 43.01) | 8.62  (3.58 to 14.62) | -4.88  (-5.59 to -4.16) | 20.01  (-78.68 to 575.44) |
| Colombia | 17.33  (13.84 to 21.22) | 13.08  (8.62 to 19.25) | -1.71  (-2.29 to -1.12) | -5.40  (-21.09 to 13.41) |
| Comoros | 136.68  (55.81 to 286.47) | 45.89  (16.61 to 105.00) | -3.93  (-4.25 to -3.62) | -2.38  (-2.89 to -1.87) |
| Congo | 59.45  (17.89 to 156.28) | 27.33  (10.53 to 61.97) | -2.67  (-2.83 to -2.51) | -3.95  (-7.14 to -0.67) |
| Cook Islands | 35.37  (13.14 to 66.45) | 21.42  (3.98 to 44.83) | -4.04  (-4.92 to -3.15) | 63.80  (-94.10 to 4450.06) |
| Costa Rica | 14.23  (10.83 to 18.41) | 7.09  (3.82 to 14.96) | -2.52  (-2.77 to -2.26) | -7.00  (-33.26 to 29.60) |
| Croatia | 45.68  (35.89 to 56.47) | 20.10  (13.32 to 32.74) | -2.81  (-3.03 to -2.59) | -4.70  (-5.38 to -4.00) |
| Cuba | 4.94  (3.90 to 6.28) | 0.94  (0.60 to 1.37) | -5.41  (-6.21 to -4.61) | -8.32  (-14.17 to -2.07) |
| Cyprus | 95.40  (29.54 to 182.74) | 19.97  (8.63 to 38.21) | -5.31  (-5.48 to -5.15) | -3.79  (-13.34 to 6.82) |
| Czechia | 36.18  (28.89 to 45.09) | 13.55  (8.78 to 20.12) | -3.10  (-3.47 to -2.72) | -8.88  (-35.32 to 28.38) |
| Côte d’Ivoire | 111.07  (45.45 to 210.77) | 47.09  (19.24 to 103.35) | -2.48  (-2.80 to -2.16) | -5.48  (-10.28 to -0.42) |
| Democratic People's Republic of Korea | 22.73  (7.78 to 54.18) | 17.08  (7.50 to 34.05) | -0.52  (-0.81 to -0.22) | -3.65  (-4.41 to -2.88) |
| Democratic Republic of the Congo | 74.19  (18.79 to 189.24) | 29.80  (11.09 to 64.06) | -2.97  (-3.29 to -2.64) | -5.64  (-10.90 to -0.06) |
| Denmark | 41.58  (33.41 to 51.13) | 4.92  (3.46 to 6.83) | -7.18  (-7.57 to -6.79) | -2.63  (-6.68 to 1.59) |
| Djibouti | 56.72  (13.72 to 160.48) | 30.77  (9.91 to 74.64) | -2.27  (-2.54 to -2.00) | -4.29  (-6.93 to -1.59) |
| Dominica | 9.73  (4.60 to 18.27) | 10.30  (4.33 to 22.00) | 0.18  (0.08 to 0.29) | 0.18  (0.03 to 0.32) |
| Dominican Republic | 14.95  (5.38 to 33.82) | 9.34  (4.21 to 17.56) | -1.13  (-1.40 to -0.86) | -1.87  (-21.29 to 22.35) |
| Ecuador | 26.91  (18.43 to 41.43) | 5.64  (3.59 to 8.71) | -5.93  (-7.17 to -4.67) | -7.31  (-32.27 to 26.84) |
| Egypt | 195.79  (95.37 to 351.52) | 42.87  (21.68 to 71.63) | -5.17  (-5.46 to -4.88) | -6.43  (-33.69 to 32.03) |
| El Salvador | 22.30  (9.13 to 40.83) | 10.25  (3.92 to 20.76) | -2.59  (-2.75 to -2.43) | -3.43  (-6.27 to -0.51) |
| Equatorial Guinea | 165.07  (77.14 to 301.01) | 36.25  (13.49 to 77.83) | -5.43  (-5.67 to -5.19) | -4.28  (-17.16 to 10.61) |
| Eritrea | 157.52  (62.91 to 384.59) | 75.09  (33.03 to 142.10) | -2.29  (-2.50 to -2.08) | -3.66  (-3.72 to -3.60) |
| Estonia | 42.92  (30.17 to 60.94) | 9.23  (6.19 to 13.48) | -5.17  (-5.93 to -4.41) | -3.06  (-12.94 to 7.94) |
| Eswatini | 58.52  (18.49 to 125.14) | 40.95  (10.52 to 114.29) | -0.96  (-1.12 to -0.80) | -1.21  (-5.95 to 3.77) |
| Ethiopia | 288.48  (114.67 to 639.98) | 70.59  (34.09 to 124.73) | -4.31  (-4.78 to -3.83) | -7.73  (-37.18 to 35.54) |
| Fiji | 29.37  (10.04 to 59.21) | 18.70  (8.39 to 33.74) | -1.59  (-1.79 to -1.40) | -2.18  (-13.73 to 10.92) |
| Finland | 54.17  (45.07 to 63.66) | 9.52  (7.22 to 12.59) | -5.30  (-5.57 to -5.03) | -5.33  (-25.38 to 20.09) |
| France | 281.77  (261.07 to 303.17) | 31.27  (24.19 to 40.90) | -6.73  (-7.51 to -5.94) | -5.25  (-18.06 to 9.56) |
| Gabon | 46.77  (17.60 to 105.61) | 23.67  (9.71 to 46.86) | -1.60  (-1.85 to -1.35) | -8.93  (-16.52 to -0.66) |
| Gambia | 75.90  (36.93 to 135.29) | 30.41  (15.51 to 51.84) | -2.87  (-3.15 to -2.59) | -5.00  (-13.35 to 4.15) |
| Georgia | 0.02  (0.01 to 0.04) | 14.95  (9.59 to 22.17) | 33.36  (26.85 to 40.21) | -3.73  (-4.77 to -2.69) |
| Germany | 223.31  (204.82 to 242.37) | 20.56  (15.34 to 27.19) | -7.75  (-7.91 to -7.59) | -5.39  (-25.31 to 19.83) |
| Ghana | 83.88  (39.70 to 144.75) | 32.52  (13.76 to 62.88) | -3.03  (-3.25 to -2.81) | -6.69  (-10.08 to -3.17) |
| Greece | 19.93  (16.21 to 23.97) | 4.27  (3.01 to 6.05) | -5.25  (-5.77 to -4.73) | -7.12  (-32.95 to 28.64) |
| Greenland | 249.00  (98.69 to 483.90) | 88.81  (48.15 to 145.20) | -3.30  (-3.53 to -3.07) | -2.66  (-55.80 to 114.39) |
| Grenada | 17.45  (12.48 to 24.21) | 0.05  (0.03 to 0.07) | -14.81  (-18.69 to -10.73) | -2.66  (-3.90 to -1.41) |
| Guam | 31.65  (12.55 to 56.05) | 20.68  (10.36 to 37.13) | -1.01  (-1.19 to -0.83) | -6.40  (-28.63 to 22.76) |
| Guatemala | 51.68  (39.31 to 71.01) | 42.99  (31.82 to 58.59) | 0.41  (-0.18 to 1.01) | -7.09  (-32.28 to 27.47) |
| Guinea | 194.54  (88.43 to 333.84) | 86.74  (37.64 to 163.33) | -2.50  (-2.80 to -2.20) | -4.83  (-5.67 to -3.98) |
| Guinea-Bissau | 166.78  (74.80 to 291.10) | 70.42  (31.69 to 127.49) | -2.42  (-2.66 to -2.17) | -6.79  (-9.01 to -4.51) |
| Guyana | 22.85  (14.31 to 33.03) | 9.35  (5.63 to 14.28) | -2.42  (-2.76 to -2.07) | -4.15  (-4.30 to -4.01) |
| Haiti | 90.22  (36.29 to 217.68) | 47.88  (18.87 to 99.88) | -2.28  (-2.46 to -2.10) | -2.07  (-7.43 to 3.61) |
| Honduras | 34.07  (13.44 to 68.62) | 11.98  (3.27 to 27.80) | -3.83  (-4.11 to -3.55) | -2.99  (-8.39 to 2.73) |
| Hungary | 47.02  (37.56 to 56.47) | 23.82  (16.18 to 35.84) | -2.61  (-2.86 to -2.36) | -6.02  (-8.22 to -3.78) |
| Iceland | 125.38  (106.77 to 144.61) | 13.54  (10.30 to 17.56) | -6.97  (-7.10 to -6.84) | -4.82  (-17.71 to 10.10) |
| India | 118.08  (55.11 to 216.15) | 36.23  (17.77 to 59.67) | -4.20  (-4.69 to -3.70) | -11.26  (-51.17 to 61.27) |
| Indonesia | 89.98  (36.24 to 167.17) | 26.03  (11.26 to 42.35) | -4.40  (-4.57 to -4.22) | -2.06  (-18.48 to 17.67) |
| Iran (Islamic Republic of) | 135.69  (68.97 to 245.34) | 12.85  (7.14 to 21.56) | -4.32  (-5.21 to -3.42) | -44.11  (-71.62 to 10.06) |
| Iraq | 140.66  (72.10 to 237.72) | 55.33  (27.63 to 93.26) | -3.44  (-3.70 to -3.17) | -5.48  (-16.68 to 7.23) |
| Ireland | 240.52  (218.11 to 264.17) | 30.39  (21.27 to 42.40) | -6.40  (-6.77 to -6.04) | -5.37  (-32.96 to 33.57) |
| Israel | 75.34  (50.07 to 98.37) | 8.38  (6.02 to 11.28) | -6.07  (-6.42 to -5.71) | -18.42  (-26.27 to -9.74) |
| Italy | 24.62  (21.76 to 27.54) | 5.23  (3.87 to 6.89) | -5.51  (-6.08 to -4.93) | -0.81  (-18.14 to 20.18) |
| Jamaica | 13.57  (10.31 to 17.83) | 3.83  (2.37 to 6.04) | -4.12  (-4.55 to -3.68) | -3.00  (-12.67 to 7.74) |
| Japan | 42.11  (38.57 to 46.02) | 11.83  (9.66 to 14.46) | -5.25  (-5.81 to -4.69) | -4.78  (-10.02 to 0.78) |
| Jordan | 62.62  (30.25 to 109.72) | 29.85  (16.78 to 48.84) | -2.02  (-2.16 to -1.89) | -2.19  (-17.48 to 15.94) |
| Kazakhstan | 44.86  (14.33 to 68.52) | 21.58  (13.20 to 34.47) | -3.13  (-3.60 to -2.66) | -5.16  (-5.69 to -4.63) |
| Kenya | 90.76  (47.19 to 146.19) | 35.62  (19.90 to 59.33) | -2.83  (-3.42 to -2.24) | -9.03  (-35.59 to 28.49) |
| Kiribati | 76.96  (23.73 to 185.84) | 31.77  (12.54 to 56.70) | -2.99  (-3.07 to -2.92) | -1.54  (-2.54 to -0.54) |
| Kuwait | 51.18  (36.31 to 67.33) | 41.97  (30.99 to 55.65) | 0.09  (-0.52 to 0.71) | -6.01  (-40.56 to 48.63) |
| Kyrgyzstan | 26.10  (16.48 to 40.84) | 7.77  (3.84 to 15.13) | -5.02  (-5.68 to -4.36) | -2.33  (-8.47 to 4.22) |
| Lao People's Democratic Republic | 134.01  (47.41 to 281.85) | 49.64  (18.83 to 96.29) | -3.52  (-4.08 to -2.95) | -2.05  (-3.12 to -0.96) |
| Latvia | 100.31  (74.34 to 131.76) | 35.75  (25.70 to 48.26) | -4.20  (-5.07 to -3.32) | -4.26  (-10.92 to 2.89) |
| Lebanon | 49.72  (20.46 to 95.19) | 26.77  (13.02 to 51.22) | -1.93  (-2.07 to -1.80) | -4.16  (-20.16 to 15.03) |
| Lesotho | 75.19  (35.16 to 139.18) | 36.28  (9.04 to 111.62) | -2.45  (-2.77 to -2.13) | 1.44  (-1.56 to 4.53) |
| Liberia | 188.24  (72.93 to 401.95) | 55.76  (23.53 to 112.99) | -4.38  (-4.69 to -4.08) | -3.10  (-8.85 to 3.01) |
| Libya | 52.42  (20.81 to 116.53) | 32.75  (14.70 to 58.95) | -1.17  (-1.50 to -0.84) | -3.00  (-9.69 to 4.18) |
| Lithuania | 21.69  (15.46 to 30.04) | 15.61  (11.33 to 21.15) | -1.94  (-2.98 to -0.89) | -6.13  (-29.46 to 24.92) |
| Luxembourg | 298.30  (268.75 to 330.84) | 28.85  (21.53 to 38.61) | -7.31  (-8.04 to -6.57) | -5.28  (-10.81 to 0.59) |
| Madagascar | 105.80  (41.23 to 243.61) | 67.34  (30.42 to 118.93) | -1.22  (-1.65 to -0.78) | -5.84  (-10.88 to -0.51) |
| Malawi | 120.14  (35.86 to 313.70) | 35.85  (12.73 to 83.19) | -4.26  (-4.66 to -3.86) | -3.67  (-6.79 to -0.45) |
| Malaysia | 19.08  (7.83 to 37.52) | 11.35  (3.95 to 22.62) | -1.57  (-1.88 to -1.26) | -5.23  (-41.84 to 54.42) |
| Maldives | 35.62  (15.78 to 65.00) | 13.71  (5.54 to 25.40) | -3.01  (-3.27 to -2.74) | -2.50  (-36.99 to 50.86) |
| Mali | 138.00  (52.35 to 239.80) | 54.67  (18.75 to 123.36) | -3.12  (-3.54 to -2.70) | -3.38  (-6.94 to 0.32) |
| Malta | 14.95  (11.73 to 19.14) | 2.77  (1.95 to 3.75) | -5.21  (-5.66 to -4.76) | -6.91  (-43.26 to 52.71) |
| Marshall Islands | 45.87  (18.03 to 86.80) | 21.98  (10.14 to 40.47) | -2.73  (-2.92 to -2.54) | -1.16  (-8.44 to 6.71) |
| Mauritania | 76.14  (31.49 to 138.45) | 34.16  (15.26 to 63.71) | -2.11  (-2.54 to -1.68) | -3.80  (-23.58 to 21.11) |
| Mauritius | 17.88  (13.06 to 23.95) | 28.44  (19.44 to 40.32) | 1.66  (0.09 to 3.24) | -8.34  (-14.88 to -1.30) |
| Mexico | 26.01  (22.85 to 30.07) | 35.93  (25.75 to 50.06) | 2.21  (1.74 to 2.68) | -7.00  (-35.67 to 34.45) |
| Micronesia (Federated States of) | 61.57  (24.77 to 111.80) | 20.51  (10.43 to 36.91) | -3.72  (-3.81 to -3.63) | -2.78  (-9.94 to 4.96) |
| Monaco | 80.10  (28.89 to 145.98) | 50.19  (13.85 to 85.47) | -3.36  (-4.12 to -2.60) | 45.73  (-90.45 to 2124.13) |
| Mongolia | 13.83  (3.55 to 37.30) | 13.92  (5.15 to 31.30) | 0.29  (0.06 to 0.53) | -1.15  (-59.64 to 142.12) |
| Montenegro | 35.43  (14.51 to 76.75) | 12.23  (2.73 to 29.04) | -3.66  (-4.04 to -3.28) | -4.78  (-8.30 to -1.12) |
| Morocco | 294.60  (149.57 to 591.36) | 57.14  (26.83 to 99.96) | -4.99  (-5.14 to -4.85) | -5.88  (-14.94 to 4.15) |
| Mozambique | 214.26  (81.05 to 400.44) | 65.44  (24.47 to 141.91) | -3.44  (-3.61 to -3.26) | -6.59  (-6.70 to -6.48) |
| Myanmar | 110.77  (42.62 to 208.76) | 50.53  (20.24 to 92.92) | -2.24  (-2.61 to -1.87) | -3.62  (-6.82 to -0.31) |
| Namibia | 40.17  (15.91 to 77.70) | 29.34  (9.40 to 63.79) | -0.73  (-0.82 to -0.63) | -1.61  (-5.48 to 2.42) |
| Nauru | 37.05  (12.24 to 77.66) | 25.60  (10.49 to 48.57) | -1.50  (-1.78 to -1.21) | -1.09  (-4.71 to 2.67) |
| Nepal | 195.92  (73.58 to 448.51) | 44.15  (21.79 to 82.07) | -5.20  (-5.77 to -4.64) | -2.77  (-4.72 to -0.78) |
| Netherlands | 112.82  (100.81 to 125.74) | 13.65  (10.29 to 17.64) | -6.05  (-6.67 to -5.42) | -5.44  (-15.12 to 5.33) |
| New Zealand | 421.75  (386.74 to 456.78) | 65.94  (51.10 to 82.19) | -6.20  (-6.51 to -5.88) | -5.75  (-62.07 to 134.20) |
| Nicaragua | 32.24  (16.00 to 57.25) | 13.75  (3.99 to 26.55) | -2.73  (-3.10 to -2.37) | -3.00  (-5.05 to -0.89) |
| Niger | 206.46  (89.31 to 399.00) | 91.74  (37.52 to 178.84) | -2.99  (-3.24 to -2.73) | -2.62  (-9.08 to 4.30) |
| Nigeria | 135.71  (64.37 to 240.62) | 110.67  (48.02 to 187.70) | -0.08  (-0.28 to 0.11) | -6.46  (-24.54 to 15.95) |
| Niue | 38.59  (14.98 to 71.80) | 85.43  (46.19 to 136.65) | -0.51  (-1.59 to 0.58) | 82.31  (-97.53 to 13348.91) |
| North Macedonia | 56.62  (13.99 to 139.50) | 13.93  (5.51 to 27.82) | -3.92  (-4.15 to -3.69) | -9.04  (-38.55 to 34.64) |
| Northern Mariana Islands | 17.69  (6.53 to 33.94) | 9.56  (4.64 to 17.57) | -1.98  (-2.09 to -1.87) | -3.88  (-9.48 to 2.07) |
| Norway | 221.48  (203.15 to 240.30) | 17.81  (14.95 to 21.24) | -7.30  (-7.65 to -6.95) | -6.16  (-53.12 to 87.86) |
| Oman | 69.56  (36.74 to 116.48) | 31.59  (16.58 to 50.63) | -1.74  (-2.29 to -1.18) | -7.02  (-9.19 to -4.79) |
| Pakistan | 288.78  (100.20 to 592.35) | 67.27  (30.32 to 126.16) | -4.08  (-4.35 to -3.82) | -14.83  (-69.11 to 134.86) |
| Palau | 41.00  (12.86 to 99.30) | 22.94  (11.02 to 40.66) | -1.93  (-1.98 to -1.89) | -1.58  (-3.51 to 0.38) |
| Palestine | 51.47  (17.84 to 108.41) | 34.44  (17.92 to 57.67) | -1.15  (-1.37 to -0.94) | -3.46  (-16.43 to 11.53) |
| Panama | 4.81  (3.77 to 6.20) | 12.86  (8.99 to 17.61) | 4.08  (3.73 to 4.44) | -5.23  (-24.03 to 18.22) |
| Papua New Guinea | 118.28  (40.71 to 228.84) | 69.53  (32.44 to 121.52) | -1.96  (-2.24 to -1.69) | -2.71  (-2.94 to -2.47) |
| Paraguay | 14.09  (2.70 to 32.52) | 7.98  (2.14 to 15.77) | -2.14  (-2.56 to -1.72) | -1.49  (-24.03 to 27.72) |
| Peru | 45.33  (19.58 to 89.81) | 9.02  (4.25 to 16.75) | -5.35  (-5.73 to -4.97) | -10.95  (-32.69 to 17.79) |
| Philippines | 60.93  (20.74 to 108.34) | 23.19  (10.06 to 38.74) | -3.31  (-3.59 to -3.02) | -3.64  (-29.41 to 31.54) |
| Poland | 46.53  (38.15 to 56.30) | 7.75  (5.11 to 11.39) | -5.90  (-6.60 to -5.18) | -2.29  (-20.47 to 20.04) |
| Portugal | 19.88  (15.82 to 24.51) | 2.07  (1.48 to 2.83) | -7.84  (-8.68 to -7.00) | -10.38  (-41.89 to 38.22) |
| Puerto Rico | 15.90  (12.03 to 20.52) | 0.00  (0.00 to 0.01) | -19.14  (-24.45 to -13.46) | -4.64  (-14.24 to 6.05) |
| Qatar | 43.03  (17.06 to 79.07) | 15.78  (7.10 to 30.55) | -3.04  (-3.12 to -2.96) | -6.92  (-25.78 to 16.74) |
| Republic of Korea | 20.76  (1.67 to 51.90) | 11.11  (1.52 to 24.80) | -1.61  (-1.86 to -1.36) | -11.69  (-23.45 to 1.87) |
| Republic of Moldova | 7.07  (4.69 to 10.07) | 23.60  (15.32 to 35.22) | 2.18  (0.72 to 3.66) | -3.16  (-4.28 to -2.03) |
| Romania | 6.75  (5.16 to 8.99) | 2.84  (1.79 to 4.30) | -2.56  (-2.91 to -2.21) | -2.90  (-14.55 to 10.34) |
| Russian Federation | 52.12  (45.71 to 59.65) | 26.40  (22.20 to 31.83) | -2.77  (-3.32 to -2.22) | 5.91  (-17.92 to 36.66) |
| Rwanda | 163.17  (72.12 to 292.45) | 33.39  (12.52 to 70.42) | -6.58  (-7.49 to -5.65) | -2.15  (-13.24 to 10.36) |
| Saint Kitts and Nevis | 31.44  (22.88 to 42.04) | 8.85  (5.65 to 13.86) | -3.66  (-4.30 to -3.01) | -2.50  (-22.80 to 23.16) |
| Saint Lucia | 67.09  (50.99 to 86.21) | 23.57  (14.98 to 35.95) | -3.15  (-3.69 to -2.62) | -3.10  (-20.88 to 18.67) |
| Saint Vincent and the Grenadines | 33.09  (23.64 to 44.51) | 4.07  (2.59 to 6.12) | -6.74  (-7.60 to -5.88) | -1.74  (-24.96 to 28.66) |
| Samoa | 52.75  (20.95 to 94.86) | 23.28  (9.60 to 50.17) | -2.54  (-2.64 to -2.45) | -2.98  (-4.24 to -1.69) |
| San Marino | 72.81  (25.69 to 127.37) | 17.38  (6.70 to 30.91) | -4.12  (-4.35 to -3.89) | -14.85  (-53.97 to 57.50) |
| Sao Tome and Principe | 96.20  (43.49 to 190.60) | 26.74  (12.10 to 48.32) | -4.30  (-4.81 to -3.79) | -4.61  (-23.66 to 19.20) |
| Saudi Arabia | 73.35  (32.15 to 137.12) | 17.79  (6.65 to 33.76) | -4.17  (-4.29 to -4.06) | -7.26  (-33.97 to 30.26) |
| Senegal | 100.55  (42.31 to 181.04) | 41.67  (15.88 to 90.29) | -2.22  (-2.59 to -1.85) | -7.50  (-8.72 to -6.27) |
| Serbia | 37.96  (15.20 to 68.61) | 13.20  (3.58 to 26.56) | -3.91  (-4.49 to -3.32) | -4.60  (-7.19 to -1.94) |
| Seychelles | 14.46  (5.49 to 25.84) | 13.90  (6.00 to 26.33) | -0.03  (-0.20 to 0.13) | 0.23  (-12.12 to 14.33) |
| Sierra Leone | 178.47  (65.28 to 392.01) | 49.77  (12.70 to 139.15) | -4.31  (-4.97 to -3.65) | -3.50  (-11.23 to 4.90) |
| Singapore | 2.20  (0.75 to 3.96) | 0.36  (0.23 to 0.56) | -4.98  (-5.47 to -4.49) | -1.70  (-60.61 to 145.33) |
| Slovakia | 26.07  (10.84 to 45.83) | 20.03  (7.81 to 37.61) | -1.13  (-1.44 to -0.82) | -5.20  (-10.33 to 0.21) |
| Slovenia | 32.43  (23.89 to 43.17) | 7.16  (4.71 to 10.91) | -4.72  (-5.02 to -4.42) | -5.62  (-9.80 to -1.24) |
| Solomon Islands | 58.42  (20.64 to 112.79) | 26.05  (12.08 to 48.89) | -2.94  (-3.08 to -2.80) | -1.73  (-3.94 to 0.52) |
| Somalia | 164.96  (77.90 to 305.01) | 102.03  (50.23 to 178.07) | -1.63  (-1.82 to -1.44) | -3.16  (-4.10 to -2.21) |
| South Africa | 45.17  (14.77 to 110.68) | 29.80  (10.29 to 77.32) | -1.17  (-1.32 to -1.02) | -5.28  (-35.05 to 38.13) |
| South Sudan | 228.98  (94.56 to 417.26) | 194.91  (90.51 to 359.28) | -0.77  (-1.09 to -0.44) | 0.33  (-9.57 to 11.31) |
| Spain | 60.26  (51.69 to 69.87) | 13.10  (9.48 to 18.08) | -5.08  (-5.39 to -4.76) | -6.21  (-26.99 to 20.48) |
| Sri Lanka | 13.49  (5.74 to 24.10) | 9.96  (3.20 to 19.23) | -0.75  (-1.01 to -0.50) | -3.50  (-13.37 to 7.51) |
| Sudan | 268.35  (129.83 to 537.38) | 110.04  (52.95 to 231.30) | -2.79  (-3.14 to -2.44) | -5.56  (-7.53 to -3.54) |
| Suriname | 18.36  (6.78 to 38.57) | 8.53  (3.99 to 16.13) | -2.90  (-3.14 to -2.67) | -3.55  (-16.95 to 12.02) |
| Sweden | 160.67  (147.69 to 174.45) | 16.85  (13.77 to 21.02) | -6.24  (-6.81 to -5.67) | -5.17  (-35.68 to 39.83) |
| Switzerland | 46.01  (37.39 to 56.00) | 5.59  (3.84 to 8.22) | -7.47  (-7.88 to -7.05) | -3.42  (-5.08 to -1.73) |
| Syrian Arab Republic | 165.52  (85.53 to 296.53) | 27.69  (9.45 to 53.38) | -5.31  (-5.98 to -4.64) | -6.78  (-29.18 to 22.70) |
| Taiwan (Province of China) | 65.19  (55.65 to 75.60) | 21.93  (15.47 to 30.04) | -4.62  (-5.31 to -3.92) | -3.19  (-22.99 to 21.72) |
| Tajikistan | 15.02  (5.25 to 34.90) | 21.04  (8.17 to 50.70) | 0.98  (0.71 to 1.26) | -3.35  (-20.54 to 17.56) |
| Thailand | 27.28  (11.98 to 47.00) | 13.36  (5.97 to 23.65) | -2.23  (-2.41 to -2.05) | -1.73  (-7.12 to 3.98) |
| Timor-Leste | 115.67  (45.37 to 286.99) | 50.27  (21.20 to 87.44) | -3.39  (-3.68 to -3.11) | -0.24  (-3.63 to 3.27) |
| Togo | 76.79  (28.90 to 146.61) | 35.18  (14.14 to 68.33) | -2.48  (-2.75 to -2.20) | -5.83  (-12.56 to 1.42) |
| Tokelau | 42.52  (17.94 to 77.77) | 160.29  (70.52 to 292.92) | -0.72  (-2.62 to 1.22) | 188.68  (-99.87 to 640084.81) |
| Tonga | 35.06  (12.98 to 62.89) | 17.23  (7.65 to 34.51) | -2.69  (-2.86 to -2.52) | -1.53  (-3.67 to 0.65) |
| Trinidad and Tobago | 54.13  (40.20 to 71.85) | 13.66  (9.32 to 19.61) | -5.38  (-6.40 to -4.35) | -1.70  (-13.51 to 11.73) |
| Tunisia | 108.48  (45.41 to 216.81) | 30.84  (16.29 to 52.96) | -3.73  (-4.11 to -3.35) | -4.45  (-19.83 to 13.88) |
| Turkmenistan | 21.97  (5.64 to 34.13) | 17.84  (9.92 to 29.55) | -0.60  (-0.79 to -0.41) | -2.74  (-4.44 to -1.02) |
| Tuvalu | 64.70  (12.99 to 179.36) | 22.20  (10.31 to 41.68) | -3.49  (-3.63 to -3.36) | -2.71  (-5.02 to -0.33) |
| Turkey | 143.25  (51.12 to 285.18) | 32.53  (17.59 to 52.82) | -4.96  (-5.30 to -4.63) | -5.88  (-20.08 to 10.83) |
| Uganda | 110.87  (45.13 to 206.13) | 43.02  (17.13 to 88.44) | -3.70  (-4.18 to -3.22) | -1.44  (-2.44 to -0.42) |
| Ukraine | 64.04  (42.41 to 94.44) | 36.38  (23.92 to 63.24) | -1.96  (-2.09 to -1.83) | -0.68  (-24.47 to 30.60) |
| United Arab Emirates | 42.44  (21.90 to 74.81) | 17.30  (8.47 to 31.11) | -2.14  (-2.51 to -1.77) | -5.68  (-27.51 to 22.71) |
| United Kingdom | 239.11  (229.37 to 250.61) | 23.11  (18.98 to 26.99) | -6.70  (-7.07 to -6.33) | -6.81  (-65.69 to 153.12) |
| United Republic of Tanzania | 127.90  (39.24 to 304.61) | 66.11  (25.50 to 130.89) | -2.22  (-2.72 to -1.71) | -7.40  (-9.07 to -5.70) |
| United States of America | 231.33  (221.53 to 240.65) | 58.70  (49.81 to 67.75) | -4.00  (-4.31 to -3.69) | -4.23  (-5.13 to -3.33) |
| United States Virgin Islands | 10.21  (4.69 to 18.53) | 4.66  (1.28 to 9.99) | -2.37  (-2.45 to -2.30) | -4.32  (-8.48 to 0.04) |
| Uruguay | 60.54  (48.80 to 74.58) | 31.14  (21.75 to 43.13) | -3.00  (-4.24 to -1.75) | -4.10  (-12.88 to 5.56) |
| Uzbekistan | 18.43  (5.88 to 28.20) | 15.13  (8.33 to 24.18) | -0.66  (-0.82 to -0.51) | -2.46  (-7.71 to 3.09) |
| Vanuatu | 45.51  (17.98 to 86.50) | 23.69  (11.28 to 44.92) | -2.17  (-2.43 to -1.91) | -2.11  (-7.13 to 3.18) |
| Venezuela (Bolivarian Republic of) | 4.94  (3.96 to 6.06) | 8.04  (5.29 to 12.40) | 1.47  (0.91 to 2.03) | -7.40  (-14.15 to -0.12) |
| Viet Nam | 53.89  (20.61 to 94.90) | 20.97  (7.75 to 41.70) | -3.18  (-3.50 to -2.87) | -1.72  (-3.34 to -0.07) |
| Yemen | 292.36  (143.62 to 508.38) | 145.29  (65.29 to 292.93) | -2.41  (-2.64 to -2.19) | -3.95  (-4.06 to -3.84) |
| Zambia | 113.45  (37.66 to 259.98) | 39.14  (13.95 to 85.91) | -4.02  (-4.69 to -3.34) | -4.49  (-14.07 to 6.16) |
| Zimbabwe | 46.07  (18.87 to 99.08) | 40.04  (14.51 to 96.21) | -0.53  (-1.02 to -0.03) | 1.59  (-19.24 to 27.79) |

ASDR= Age standardized disability-adjusted life years rate, SIDS= sudden infant death syndrome, EAPC= estimated annual percentage changes, UI = uncertainty intervals, CI= confidence interval





Figure S2. The proportion of DALYs attributable to risk factors of SIDS for 21 geographic regions in 2021.

DALYs= disability-adjusted life years, SIDS= sudden infant death syndrome, SDI= socio-demographic index

Table S4. The proportion of DALYs attributable to risk factors of SIDS for global, five SDI regions, and 21 geographic regions in 2021

| Location | Risk facto | Proportion | 95% LUI | 95% UUI |
| --- | --- | --- | --- | --- |
| Global | Ambient particulate matter pollution | 0.71 | 0.46 | 1.00 |
|  | Household air pollution from solid fuels | 1.62 | 1.19 | 2.10 |
|  | Short gestation | 3.11 | 2.51 | 3.73 |
|  | Low birth weight | 7.09 | 5.89 | 8.45 |
| **SDI** |  |  |  |  |
| Low SDI | Ambient particulate matter pollution | 0.45 | 0.24 | 0.77 |
|  | Short gestation | 3.05 | 2.22 | 3.91 |
|  | Low birth weight | 6.46 | 4.77 | 8.24 |
|  | Household air pollution from solid fuels | 2.11 | 1.53 | 2.77 |
| Low-middle SDI | Ambient particulate matter pollution | 0.90 | 0.55 | 1.38 |
|  | Short gestation | 3.60 | 2.93 | 4.42 |
|  | Low birth weight | 7.80 | 6.44 | 9.35 |
|  | Household air pollution from solid fuels | 1.70 | 1.17 | 2.31 |
| Middle SDI | Ambient particulate matter pollution | 1.37 | 0.85 | 1.83 |
|  | Short gestation | 3.37 | 2.58 | 4.17 |
|  | Low birth weight | 9.22 | 7.46 | 10.9 |
|  | Household air pollution from solid fuels | 0.67 | 0.25 | 1.32 |
| High-middle SDI | Ambient particulate matter pollution | 0.94 | 0.66 | 1.22 |
|  | Short gestation | 1.72 | 1.28 | 2.20 |
|  | Low birth weight | 6.72 | 5.74 | 7.81 |
|  | Household air pollution from solid fuels | 0.03 | 0.00 | 0.20 |
| High SDI | Ambient particulate matter pollution | 0.31 | 0.23 | 0.40 |
|  | Short gestation | 1.34 | 1.13 | 1.56 |
|  | Low birth weight | 4.46 | 4.17 | 4.76 |
|  | Household air pollution from solid fuels | 0.00 | 0.00 | 0.00 |
| **Region** |  |  |  |  |
| Andean Latin America | Ambient particulate matter pollution | 1.64 | 0.68 | 2.98 |
|  | Household air pollution from solid fuels | 0.55 | 0.12 | 1.41 |
|  | Low birth weight | 10.82 | 7.94 | 14.4 |
|  | Short gestation | 2.52 | 1.28 | 4.11 |
| Australasia | Ambient particulate matter pollution | 0.22 | 0.03 | 0.48 |
|  | Household air pollution from solid fuels | 0.00 | 0.00 | 0.00 |
|  | Low birth weight | 4.00 | 3.29 | 4.85 |
|  | Short gestation | 1.21 | 0.84 | 1.68 |
| Caribbean | Ambient particulate matter pollution | 0.54 | 0.25 | 0.92 |
|  | Household air pollution from solid fuels | 3.68 | 1.77 | 6.35 |
|  | Low birth weight | 11.51 | 7.35 | 16.94 |
|  | Short gestation | 6.76 | 4.04 | 10.22 |
| Central Asia | Ambient particulate matter pollution | 1.08 | 0.57 | 1.76 |
|  | Household air pollution from solid fuels | 0.48 | 0.21 | 0.91 |
|  | Low birth weight | 7.10 | 5.61 | 8.32 |
|  | Short gestation | 1.74 | 1.20 | 2.29 |
| Central Europe | Ambient particulate matter pollution | 0.53 | 0.30 | 0.75 |
|  | Household air pollution from solid fuels | 0.04 | 0.00 | 0.19 |
|  | Low birth weight | 4.62 | 3.75 | 5.48 |
|  | Short gestation | 0.34 | 0.20 | 0.54 |
| Central Latin America | Ambient particulate matter pollution | 1.12 | 0.58 | 1.64 |
|  | Household air pollution from solid fuels | 0.59 | 0.24 | 1.20 |
|  | Low birth weight | 12.17 | 10.21 | 14.28 |
|  | Short gestation | 2.63 | 1.97 | 3.42 |
| Central Sub-Saharan Africa | Ambient particulate matter pollution | 0.39 | 0.14 | 0.86 |
|  | Household air pollution from solid fuels | 1.60 | 0.79 | 2.70 |
|  | Low birth weight | 5.89 | 3.25 | 9.01 |
|  | Short gestation | 2.05 | 1.04 | 3.49 |
| East Asia | Ambient particulate matter pollution | 0.99 | 0.56 | 1.37 |
|  | Household air pollution from solid fuels | 0.39 | 0.14 | 0.87 |
|  | Low birth weight | 5.45 | 4.37 | 6.53 |
|  | Short gestation | 0.42 | 0.24 | 0.65 |
| Eastern Europe | Ambient particulate matter pollution | 0.39 | 0.27 | 0.54 |
|  | Household air pollution from solid fuels | 0.02 | 0.00 | 0.08 |
|  | Low birth weight | 5.05 | 4.49 | 5.46 |
|  | Short gestation | 1.38 | 1.15 | 1.61 |
| Eastern Sub-Saharan Africa | Ambient particulate matter pollution | 0.19 | 0.11 | 0.32 |
|  | Household air pollution from solid fuels | 2.37 | 1.64 | 3.25 |
|  | Low birth weight | 6.30 | 4.50 | 8.36 |
|  | Short gestation | 2.90 | 1.98 | 3.94 |
| High-income Asia Pacific | Ambient particulate matter pollution | 0.27 | 0.17 | 0.44 |
|  | Household air pollution from solid fuels | 0.00 | 0.00 | 0.00 |
|  | Low birth weight | 2.81 | 2.31 | 3.37 |
|  | Short gestation | 0.76 | 0.56 | 1.00 |
| High-income North America | Ambient particulate matter pollution | 0.20 | 0.13 | 0.28 |
|  | Household air pollution from solid fuels | 0.00 | 0.00 | 0.00 |
|  | Low birth weight | 4.29 | 3.94 | 4.69 |
|  | Short gestation | 1.58 | 1.34 | 1.82 |
| North Africa and Middle East | Ambient particulate matter pollution | 1.10 | 0.76 | 1.59 |
|  | Household air pollution from solid fuels | 0.85 | 0.48 | 1.38 |
|  | Low birth weight | 6.47 | 4.94 | 8.33 |
|  | Short gestation | 2.71 | 1.89 | 3.76 |
| Oceania | Ambient particulate matter pollution | 0.29 | 0.06 | 0.8 |
|  | Household air pollution from solid fuels | 2.89 | 1.19 | 5.93 |
|  | Low birth weight | 8.23 | 4.08 | 15.43 |
|  | Short gestation | 4.23 | 2.08 | 7.85 |
| South Asia | Ambient particulate matter pollution | 0.88 | 0.50 | 1.46 |
|  | Household air pollution from solid fuels | 1.94 | 1.34 | 2.70 |
|  | Low birth weight | 8.01 | 6.02 | 10.12 |
|  | Short gestation | 4.35 | 3.22 | 5.51 |
| Southeast Asia | Ambient particulate matter pollution | 1.43 | 0.56 | 2.53 |
|  | Household air pollution from solid fuels | 2.21 | 1.00 | 3.88 |
|  | Low birth weight | 14.06 | 7.6 | 19.18 |
|  | Short gestation | 5.64 | 2.94 | 8.04 |
| Southern Latin America | Ambient particulate matter pollution | 0.81 | 0.21 | 1.58 |
|  | Household air pollution from solid fuels | 0.02 | 0.00 | 0.16 |
|  | Low birth weight | 7.81 | 5.64 | 10.49 |
|  | Short gestation | 1.15 | 0.48 | 2.13 |
| Southern Sub-Saharan Africa | Ambient particulate matter pollution | 0.70 | 0.34 | 1.15 |
|  | Household air pollution from solid fuels | 1.11 | 0.49 | 1.98 |
|  | Low birth weight | 7.03 | 4.30 | 9.98 |
|  | Short gestation | 2.67 | 1.53 | 3.93 |
| Tropical Latin America | Ambient particulate matter pollution | 0.64 | 0.34 | 0.97 |
|  | Household air pollution from solid fuels | 0.20 | 0.06 | 0.41 |
|  | Low birth weight | 9.34 | 7.87 | 11.03 |
|  | Short gestation | 3.88 | 3.21 | 4.68 |
| Western Europe | Ambient particulate matter pollution | 0.36 | 0.18 | 0.59 |
|  | Household air pollution from solid fuels | 0.00 | 0.00 | 0.00 |
|  | Low birth weight | 5.09 | 4.43 | 5.93 |
|  | Short gestation | 0.80 | 0.52 | 1.10 |
| Western Sub-Saharan Africa | Ambient particulate matter pollution | 0.59 | 0.26 | 1.06 |
|  | Household air pollution from solid fuels | 1.81 | 1.09 | 2.63 |
|  | Low birth weight | 6.08 | 4.12 | 8.37 |
|  | Short gestation | 2.62 | 1.60 | 3.76 |

DALYs= disability-adjusted life years, SIDS= sudden infant death syndrome, SDI= socio-demographic index, LUI= lower uncertainty intervals. UUI= upper uncertainty intervals

Table S5. ASDR of SIDS with frontier analysis across all countries and territories in 2021

| Location | SDI | ASDR | Frontier ASDR | Effective difference |
| --- | --- | --- | --- | --- |
| Afghanistan | 0.337200 | 139.978779 | 33.522515 | 106.46 |
| Albania | 0.706850 | 18.072612 | 0.000463 | 18.07 |
| Algeria | 0.659501 | 38.509382 | 0.000941 | 38.51 |
| American Samoa | 0.723728 | 19.846557 | 0.000436 | 19.85 |
| Andorra | 0.869444 | 7.075228 | 0.000371 | 7.07 |
| Angola | 0.453722 | 49.898808 | 14.223980 | 35.67 |
| Antigua and Barbuda | 0.749887 | 0.000318 | 0.000318 | 0.00 |
| Argentina | 0.723123 | 33.703432 | 0.000441 | 33.70 |
| Armenia | 0.701833 | 2.482132 | 0.000461 | 2.48 |
| Australia | 0.844253 | 15.776552 | 0.000363 | 15.78 |
| Austria | 0.853837 | 17.509424 | 0.000370 | 17.51 |
| Azerbaijan | 0.694851 | 15.146292 | 0.000455 | 15.15 |
| Bahamas | 0.805021 | 25.902302 | 0.000370 | 25.90 |
| Bahrain | 0.753043 | 21.706143 | 0.000372 | 21.71 |
| Bangladesh | 0.492421 | 54.403555 | 10.912763 | 43.49 |
| Barbados | 0.746749 | 9.729612 | 0.000393 | 9.73 |
| Belarus | 0.784485 | 2.618858 | 0.000359 | 2.62 |
| Belgium | 0.853654 | 21.812953 | 0.000377 | 21.81 |
| Belize | 0.610229 | 26.200977 | 2.362688 | 23.84 |
| Benin | 0.373487 | 44.668913 | 26.227044 | 18.44 |
| Bermuda | 0.821365 | 17.956259 | 0.000370 | 17.96 |
| Bhutan | 0.473062 | 34.617630 | 11.567048 | 23.05 |
| Bolivia (Plurinational State of) | 0.599011 | 18.445148 | 2.499770 | 15.95 |
| Bosnia and Herzegovina | 0.723078 | 17.037632 | 0.000439 | 17.04 |
| Botswana | 0.642722 | 25.103259 | 0.001752 | 25.10 |
| Brazil | 0.653044 | 6.298492 | 0.000871 | 6.30 |
| Brunei Darussalam | 0.810234 | 14.277065 | 0.000369 | 14.28 |
| Bulgaria | 0.768151 | 6.828530 | 0.000372 | 6.83 |
| Burkina Faso | 0.285118 | 67.299689 | 37.495298 | 29.80 |
| Burundi | 0.289374 | 33.689597 | 33.689597 | 0.00 |
| Cabo Verde | 0.533535 | 25.615907 | 5.647308 | 19.97 |
| Cambodia | 0.473621 | 29.921221 | 11.546936 | 18.37 |
| Cameroon | 0.479691 | 52.247020 | 11.345523 | 40.90 |
| Canada | 0.873171 | 10.330798 | 0.000361 | 10.33 |
| Central African Republic | 0.309168 | 68.191972 | 34.305410 | 33.89 |
| Chad | 0.240436 | 115.164908 | 65.169342 | 50.00 |
| Chile | 0.771515 | 13.256625 | 0.000365 | 13.26 |
| China | 0.721630 | 8.616254 | 0.000446 | 8.62 |
| Colombia | 0.655443 | 13.081344 | 0.000459 | 13.08 |
| Comoros | 0.475979 | 45.892994 | 11.379597 | 34.51 |
| Congo | 0.583075 | 27.331792 | 2.638529 | 24.69 |
| Cook Islands | 0.779110 | 21.417431 | 0.000366 | 21.42 |
| Costa Rica | 0.700340 | 7.088272 | 0.000460 | 7.09 |
| Croatia | 0.798341 | 20.097067 | 0.000363 | 20.10 |
| Cuba | 0.668730 | 0.935253 | 0.000464 | 0.93 |
| Cyprus | 0.835631 | 19.970889 | 0.000369 | 19.97 |
| Czechia | 0.828450 | 13.553892 | 0.000365 | 13.55 |
| Côte d’Ivoire | 0.425942 | 47.094717 | 19.669570 | 27.43 |
| Democratic People's Republic of Korea | 0.569855 | 17.083868 | 2.795363 | 14.29 |
| Democratic Republic of the Congo | 0.383180 | 29.798330 | 21.874264 | 7.92 |
| Denmark | 0.896424 | 4.918478 | 0.000370 | 4.92 |
| Djibouti | 0.487958 | 30.765483 | 11.137913 | 19.63 |
| Dominica | 0.746967 | 10.302289 | 0.000382 | 10.30 |
| Dominican Republic | 0.619388 | 9.343291 | 0.941747 | 8.40 |
| Ecuador | 0.661017 | 5.635215 | 0.000461 | 5.63 |
| Egypt | 0.606787 | 42.872418 | 2.447367 | 40.43 |
| El Salvador | 0.563775 | 10.254702 | 2.994589 | 7.26 |
| Equatorial Guinea | 0.657857 | 36.254200 | 0.000701 | 36.25 |
| Eritrea | 0.403864 | 75.091643 | 21.517739 | 53.57 |
| Estonia | 0.844918 | 9.230532 | 0.000371 | 9.23 |
| Eswatini | 0.585460 | 40.946703 | 2.572345 | 38.37 |
| Ethiopia | 0.358823 | 70.594654 | 32.385127 | 38.21 |
| Fiji | 0.675052 | 18.703459 | 0.000458 | 18.70 |
| Finland | 0.859831 | 9.524314 | 0.000382 | 9.52 |
| France | 0.838365 | 31.270664 | 0.000369 | 31.27 |
| Gabon | 0.634691 | 23.669887 | 0.062622 | 23.61 |
| Gambia | 0.409714 | 30.411360 | 21.047183 | 9.36 |
| Georgia | 0.732474 | 14.945240 | 0.000422 | 14.94 |
| Germany | 0.902957 | 20.562147 | 0.000363 | 20.56 |
| Ghana | 0.564930 | 32.522849 | 2.817320 | 29.71 |
| Greece | 0.791854 | 4.274132 | 0.000369 | 4.27 |
| Greenland | 0.826210 | 88.810156 | 0.000370 | 88.81 |
| Grenada | 0.668993 | 0.045212 | 0.000461 | 0.04 |
| Guam | 0.803982 | 20.678222 | 0.000379 | 20.68 |
| Guatemala | 0.539972 | 42.990197 | 5.381696 | 37.61 |
| Guinea | 0.336401 | 86.742404 | 34.012099 | 52.73 |
| Guinea-Bissau | 0.353110 | 70.423132 | 32.665036 | 37.76 |
| Guyana | 0.650812 | 9.351510 | 0.000880 | 9.35 |
| Haiti | 0.448278 | 47.875348 | 14.743451 | 33.13 |
| Honduras | 0.513037 | 11.982211 | 10.268948 | 1.71 |
| Hungary | 0.790755 | 23.816811 | 0.000370 | 23.82 |
| Iceland | 0.876362 | 13.535428 | 0.000366 | 13.54 |
| India | 0.575402 | 36.228011 | 2.705758 | 33.52 |
| Indonesia | 0.656868 | 26.033617 | 0.000771 | 26.03 |
| Iran (Islamic Republic of) | 0.697207 | 12.845816 | 0.000462 | 12.85 |
| Iraq | 0.662626 | 55.332366 | 0.000461 | 55.33 |
| Ireland | 0.873754 | 30.389563 | 0.000369 | 30.39 |
| Israel | 0.809012 | 8.384648 | 0.000364 | 8.38 |
| Italy | 0.805774 | 5.230065 | 0.000372 | 5.23 |
| Jamaica | 0.683263 | 3.831610 | 0.000455 | 3.83 |
| Japan | 0.871242 | 11.834972 | 0.000363 | 11.83 |
| Jordan | 0.725307 | 29.852721 | 0.000437 | 29.85 |
| Kazakhstan | 0.725144 | 21.575345 | 0.000438 | 21.57 |
| Kenya | 0.523768 | 35.620189 | 5.623695 | 30.00 |
| Kiribati | 0.527187 | 31.774634 | 6.738673 | 25.04 |
| Kuwait | 0.846651 | 41.968508 | 0.000363 | 41.97 |
| Kyrgyzstan | 0.603979 | 7.767922 | 2.460901 | 5.31 |
| Lao People's Democratic Republic | 0.489136 | 49.641886 | 10.856473 | 38.79 |
| Latvia | 0.830664 | 35.751179 | 0.000371 | 35.75 |
| Lebanon | 0.744746 | 26.770503 | 0.000385 | 26.77 |
| Lesotho | 0.510393 | 36.275653 | 10.311416 | 25.96 |
| Liberia | 0.352442 | 55.755544 | 32.746662 | 23.01 |
| Libya | 0.725771 | 32.754018 | 0.000434 | 32.75 |
| Lithuania | 0.856484 | 15.614901 | 0.000370 | 15.61 |
| Luxembourg | 0.884429 | 28.849618 | 0.000370 | 28.85 |
| Madagascar | 0.400247 | 67.343741 | 21.480999 | 45.86 |
| Malawi | 0.384554 | 35.854419 | 21.546563 | 14.31 |
| Malaysia | 0.742524 | 11.348581 | 0.000396 | 11.35 |
| Maldives | 0.650887 | 13.705996 | 0.000773 | 13.71 |
| Mali | 0.268580 | 54.666621 | 48.670280 | 6.00 |
| Malta | 0.801585 | 2.768416 | 0.000366 | 2.77 |
| Marshall Islands | 0.574091 | 21.982677 | 2.790738 | 19.19 |
| Mauritania | 0.498945 | 34.163546 | 10.619082 | 23.54 |
| Mauritius | 0.718260 | 28.441762 | 0.000455 | 28.44 |
| Mexico | 0.664575 | 35.929977 | 0.000461 | 35.93 |
| Micronesia (Federated States of) | 0.587535 | 20.510420 | 2.581318 | 17.93 |
| Monaco | 0.908263 | 50.192602 | 0.000370 | 50.19 |
| Mongolia | 0.617622 | 13.920128 | 1.326397 | 12.59 |
| Montenegro | 0.795801 | 12.231387 | 0.000369 | 12.23 |
| Morocco | 0.562698 | 57.136596 | 2.958336 | 54.18 |
| Mozambique | 0.326463 | 65.436510 | 34.781279 | 30.66 |
| Myanmar | 0.533901 | 50.528423 | 5.758519 | 44.77 |
| Namibia | 0.617565 | 29.343200 | 1.354978 | 27.99 |
| Nauru | 0.625178 | 25.596034 | 0.700980 | 24.90 |
| Nepal | 0.433175 | 44.145697 | 18.933960 | 25.21 |
| Netherlands | 0.888464 | 13.652386 | 0.000366 | 13.65 |
| New Zealand | 0.849442 | 65.943315 | 0.000366 | 65.94 |
| Nicaragua | 0.523958 | 13.746440 | 6.058152 | 7.69 |
| Niger | 0.168073 | 91.736052 | 91.736052 | 0.00 |
| Nigeria | 0.503391 | 110.667889 | 10.386314 | 100.28 |
| Niue | 0.726222 | 85.427163 | 0.000431 | 85.43 |
| North Macedonia | 0.750630 | 13.928125 | 0.000371 | 13.93 |
| Northern Mariana Islands | 0.771535 | 9.555218 | 0.000372 | 9.55 |
| Norway | 0.916133 | 17.812159 | 0.000367 | 17.81 |
| Oman | 0.773392 | 31.589622 | 0.000371 | 31.59 |
| Pakistan | 0.504029 | 67.265934 | 10.369319 | 56.90 |
| Palau | 0.754047 | 22.938623 | 0.000359 | 22.94 |
| Palestine | 0.631012 | 34.437214 | 0.036831 | 34.40 |
| Panama | 0.708865 | 12.858148 | 0.000457 | 12.86 |
| Papua New Guinea | 0.417797 | 69.533003 | 20.259869 | 49.27 |
| Paraguay | 0.635718 | 7.981409 | 0.049737 | 7.93 |
| Peru | 0.662054 | 9.019672 | 0.000463 | 9.02 |
| Philippines | 0.651219 | 23.190109 | 0.000971 | 23.19 |
| Poland | 0.812043 | 7.750705 | 0.000369 | 7.75 |
| Portugal | 0.744152 | 2.074963 | 0.000388 | 2.07 |
| Puerto Rico | 0.825526 | 0.004679 | 0.000360 | 0.00 |
| Qatar | 0.846861 | 15.778047 | 0.000369 | 15.78 |
| Republic of Korea | 0.886675 | 11.109532 | 0.000372 | 11.11 |
| Republic of Moldova | 0.732215 | 23.595622 | 0.000421 | 23.60 |
| Romania | 0.768454 | 2.835711 | 0.000365 | 2.84 |
| Russian Federation | 0.808536 | 26.395285 | 0.000373 | 26.39 |
| Rwanda | 0.435589 | 33.389679 | 17.946899 | 15.44 |
| Saint Kitts and Nevis | 0.754987 | 8.852260 | 0.000365 | 8.85 |
| Saint Lucia | 0.672510 | 23.570579 | 0.000459 | 23.57 |
| Saint Vincent and the Grenadines | 0.637196 | 4.068197 | 0.004409 | 4.06 |
| Samoa | 0.593393 | 23.279402 | 2.490562 | 20.79 |
| San Marino | 0.888005 | 17.382348 | 0.000365 | 17.38 |
| Sao Tome and Principe | 0.505414 | 26.739279 | 10.321545 | 16.42 |
| Saudi Arabia | 0.815143 | 17.789057 | 0.000381 | 17.79 |
| Senegal | 0.408054 | 41.665371 | 21.508020 | 20.16 |
| Serbia | 0.792416 | 13.200029 | 0.000364 | 13.20 |
| Seychelles | 0.730151 | 13.902361 | 0.000425 | 13.90 |
| Sierra Leone | 0.358666 | 49.774220 | 32.602125 | 17.17 |
| Singapore | 0.856098 | 0.364272 | 0.000371 | 0.36 |
| Slovakia | 0.810611 | 20.031731 | 0.000371 | 20.03 |
| Slovenia | 0.842431 | 7.162205 | 0.000374 | 7.16 |
| Solomon Islands | 0.429360 | 26.045058 | 19.381081 | 6.66 |
| Somalia | 0.077688 | 102.031599 | 102.031599 | 0.00 |
| South Africa | 0.679627 | 29.802234 | 0.000460 | 29.80 |
| South Sudan | 0.278371 | 194.911363 | 42.502271 | 152.41 |
| Spain | 0.769284 | 13.103212 | 0.000371 | 13.10 |
| Sri Lanka | 0.701535 | 9.961124 | 0.000461 | 9.96 |
| Sudan | 0.541950 | 110.036119 | 5.213694 | 104.82 |
| Suriname | 0.633666 | 8.525903 | 0.010958 | 8.51 |
| Sweden | 0.886880 | 16.851929 | 0.000373 | 16.85 |
| Switzerland | 0.933059 | 5.593100 | 0.000367 | 5.59 |
| Syrian Arab Republic | 0.623004 | 27.686374 | 0.948714 | 26.74 |
| Taiwan (Province of China) | 0.874747 | 21.934314 | 0.000372 | 21.93 |
| Tajikistan | 0.541511 | 21.044767 | 6.014954 | 15.03 |
| Thailand | 0.682548 | 13.364857 | 0.000456 | 13.36 |
| Timor-Leste | 0.444668 | 50.270188 | 15.856236 | 34.41 |
| Togo | 0.408534 | 35.179296 | 20.955112 | 14.22 |
| Tokelau | 0.686426 | 160.286940 | 0.000462 | 160.29 |
| Tonga | 0.626350 | 17.234158 | 0.680670 | 16.55 |
| Trinidad and Tobago | 0.768763 | 13.662086 | 0.000363 | 13.66 |
| Tunisia | 0.682432 | 30.842791 | 0.000464 | 30.84 |
| Turkey | 0.712693 | 32.526901 | 0.000460 | 32.53 |
| Turkmenistan | 0.682161 | 17.836011 | 0.000463 | 17.84 |
| Tuvalu | 0.576621 | 22.199036 | 2.720720 | 19.48 |
| Uganda | 0.423261 | 43.018148 | 20.048209 | 22.97 |
| Ukraine | 0.760774 | 36.379641 | 0.000367 | 36.38 |
| United Arab Emirates | 0.849318 | 17.302412 | 0.000368 | 17.30 |
| United Kingdom | 0.859000 | 23.105113 | 0.000368 | 23.10 |
| United Republic of Tanzania | 0.446568 | 66.109826 | 14.692694 | 51.42 |
| United States of America | 0.862448 | 58.698904 | 0.000365 | 58.70 |
| United States Virgin Islands | 0.821831 | 4.662584 | 0.000363 | 4.66 |
| Uruguay | 0.719283 | 31.139633 | 0.000454 | 31.14 |
| Uzbekistan | 0.662622 | 15.133115 | 0.000461 | 15.13 |
| Vanuatu | 0.473101 | 23.687568 | 11.638000 | 12.05 |
| Venezuela (Bolivarian Republic of) | 0.596513 | 8.035510 | 2.514330 | 5.52 |
| Viet Nam | 0.627934 | 20.969757 | 0.657010 | 20.31 |
| Yemen | 0.450376 | 145.286859 | 14.164587 | 131.12 |
| Zambia | 0.505949 | 39.137547 | 10.412943 | 28.72 |
| Zimbabwe | 0.473819 | 40.035282 | 11.581686 | 28.45 |

ASDR= Age standardized disability-adjusted life years rate, SIDS= sudden infant death syndrome, SDI= socio-demographic index, LUI= lower uncertainty intervals. UUI= upper uncertainty intervals
